# Comprehensive analysis of 2097 patients with dystrophinopathy based on a database from 2011 to 2021

**DOI:** 10.1186/s13023-024-03217-7

**Published:** 2024-08-24

**Authors:** Lei Zhao, Yiyun Shi, Chaoping Hu, Shuizhen Zhou, Hui Li, Lifeng Zhang, Chuang Qian, Yiyao Zhou, Yi Wang, Xihua Li

**Affiliations:** 1 Department of Neurology, Children’s Hospital of Fudan University, No.399, Wanyuan Road, Minhang District, Shanghai, 201102 China; 2 Department of Rehabilitation, Children’s Hospital of Fudan University, Shanghai, China; 3 Pediatric Heart Center, Children’s Hospital of Fudan University, Shanghai, China; 4 Department of Orthopedics, Children’s Hospital of Fudan University, Shanghai, China; 5 Department of Clinical Nutrition, Children’s Hospital of Fudan University, Shanghai, China

**Keywords:** Dystrophinopathy, Duchenne and Becker muscular dystrophies, Patient management, Genetic diagnosis, Natural history

## Abstract

**Background:**

An increasing number of clinical trials for new therapeutic strategies are underway or being considered for dystrophinopathy. Having detailed data on the natural progression of this condition is crucial for assessing the effectiveness of new drugs. However, there’s a lack of data regarding the long-term data on the natural course and how it’s managed in China. In this study, we offer a comprehensive overview of clinical and molecular findings, as well as treatment outcomes in the Chinese population.

**Methods:**

Institutional data on all patients with dystrophinopathy from August 2011 to August 2021 were retrospectively reviewed. The data included geographic distribution, age at diagnosis, molecular findings, and treatment options, such as corticosteroids, cardiac interventions, and clinical outcomes.

**Results:**

In total, 2097 patients with dystrophinopathy, including 1703 cases of Duchenne muscular dystrophy (DMD), 311 cases of Becker muscular dystrophy (BMD), 46 cases of intermediate muscular dystrophy (IMD), and 37 cases categorized as “pending” (individuals with an undetermined phenotype), were registered in the Children’s Hospital of Fudan University database for dystrophinopathy from August 2011 to August 2021. The spectrum of identified variants included exonic deletions (66.6%), exonic duplications (10.7%), nonsense variants (10.3%), splice-site variants (4.5%), small deletions (3.5%), small insertions/duplications (1.8%), and missense variants (0.9%). Four deep intronic variants and two inversion variants were identified. Regarding treatment, glucocorticoids were administered to 54.4% of DMD patients and 39.1% of IMD patients. The median age at loss of ambulation was 2.5 years later in DMD patients who received glucocorticoid treatment. Overall, one cardiac medicine at least was prescribed to 7.4% of DMD patients, 8.3% of IMD patients, and 2.6% of BMD patients. Additionally, ventilator support was required by four DMD patients. Eligibility for exon skipping therapy was found in 55.3% of DMD patients, with 12.9%, 10%, and 9.6% of these patients being eligible for skipping exons 51, 53, and 45, respectively.

**Conclusions:**

This is one of the largest studies to have evaluated the natural history of dystrophinopathy in China, which is particularly conducive to the recruitment of eligible patients for clinical trials and the provision of real-world data to support drug development.

**Supplementary Information:**

The online version contains supplementary material available at 10.1186/s13023-024-03217-7.

## Background

Duchenne and Becker muscular dystrophies, collectively known as dystrophinopathies, are X-linked recessive disorders caused by mutations in the dystrophin gene, resulting in either absent or insufficient functional dystrophin, a crucial cytoskeletal protein essential for myofiber strength, stability, and function. Duchenne muscular dystrophy (DMD; OMIM 310,200) is the most common form, affecting approximately 1 in 3600–6300 live male births worldwide and 1 in 4560 live male births in China [1–3]. DMD shows a severe phenotype characterized by early onset of initial motor symptoms, typically occurring before the 5th birthday, including delayed walking, frequent falls, and difficulty in running and climbing stairs. While most children with DMD show improvement in strength and motor function by the age of 5 years, they still lag behind their peers. However, a decline in function becomes evident around the age of 7 years, and most DMD patients become wheelchair dependent between 10 and 12 years of age. Even with optimal care, the majority of patients with DMD succumb to cardiac and/or respiratory failure between the ages of 20 and 40 years. Milder allelic forms, including intermediate muscular dystrophy (IMD) and Becker muscular dystrophy (BMD), lead to the loss of ambulation at 13–16 years or beyond 16 years, respectively. Becker muscular dystrophy is associated with less severe degeneration in other body systems [1, 4].

China stands out as one of the countries with a high prevalence of dystrophinopathies [3, 5–7]. In 2015, a study of 229 children with DMD/BMD in East China was published [5]. The study aimed to comprehensively analyze various aspects of the disease, including geographic distribution, age at diagnosis, distribution of pathogenic genetic variants, genotype/phenotype correlations, family history, walking ability, acceptance of corticosteroid treatment, and cardiorespiratory function. Among the participants, it was noted that 23.1% had a family history of the disease, emphasizing the importance of genetic counseling and screening for at-risk family members. Another concerning discovery was that only 26.3% of the DMD patients in the study received corticosteroids, and as many as 17.5% of children with DMD lost the ability to walk. Corticosteroids have been scientifically proven to slow the progression of muscle weakness in children with DMD. The low percentage suggests inadequate awareness or access to appropriate medical interventions in China. Furthermore, the study uncovered that a significant proportion of individuals with DMD and BMD did not undergo regular monitoring for their cardiac function (approximately 50%) and respiratory function (approximately 80%), which is essential for recognizing and managing cardiorespiratory complications. These findings underscore the urgent need for increased awareness of the disease. Additionally, the study revealed a concerning reality: a considerable number of children who experienced delayed diagnosis resided in rural regions. This highlights potential challenges in accessing healthcare services and resources for children living in remote areas. It’s essential to recognize that children from low-income families may face additional barriers due to financial constraints when covering the costs of diagnosis and treatment. Consequently, many families are forced to forego further medical evaluations and comprehensive treatment plans, exacerbating the challenges faced by researchers studying the natural history of DMD, professionals providing genetic counseling, and clinicians conducting DMD-related clinical research in China. The study also observed that China’s scientific research funding was predominantly allocated to common diseases, with limited funding allocated to rare diseases such as DMD over several years. Consequently, medical research on DMD has lagged behind that of developed countries, posing a public health concern in the field of rare diseases in China.

In the past eight years, significant progress has been achieved in the medical approach to managing DMD. Although there is currently no cure, ongoing developments in DMD drugs aim to slow down disease progression. These drugs include eteplirsen (Exondys 51) [8], golodirsen (Vyondys 53) [9], viltolarsen (Viltepso) [10], casimersen (Amondys 45) [11], delandistrogene moxeparvovec-rokl (Elevidys) [12], and gene therapy phase III clinical trials (ClinicalTrials.gov, Identifier: NCT03362502, NCT04281485, NCT05429372, NCT03179631, and NCT01247207) are taking place globally. These technological advancements have significantly enhanced DMD diagnosis and treatment in China, benefiting patient care. They also promote the exchange of scientific knowledge concerning DMD gene therapy, encourage participation in multinational clinical trials for new DMD-targeting drugs (ClinicalTrials.gov, Identifier: NCT03179631 and NCT04956289), facilitate the study of DMD’s natural history (ClinicalTrials.gov, Identifier: NCT03760029), and bolster both fundamental research and drug development efforts for DMD within China [13, 14]. These endeavors formed the basis for the inclusion of DMD in China’s First List of Rare Diseases in 2018 [15]. However, China’s DMD/BMD research data lags behind that of Japan (http://remudy.ncnp.go.jp), the United States (http://www.duchenneregistry.org), and the European Union (http://treat-nmd.org/resources-support/patient-registries/), all of which have established DMD/BMD registration platforms. Natural history studies in these countries played a pivotal role in shaping standardized care and generating real-world evidence [16–25]. Such natural history data have also served as control data in various clinical trials of new DMD drugs [26–28]. Despite some efforts to establish registry systems [29] and conducting studies on the Chinese population [30–32], comprehensive data on how corticosteroid treatment affects the motor function of individuals with DMD and their current management of cardiopulmonary function remain lacking. Therefore, it is imperative to establish a systematic and comprehensive dystrophinopathy database encompassing geographic distribution, clinical and genetic data, and treatment information to unveil the characteristics of the DMD population in China. This database can facilitate the expansion of health insurance coverage for rare diseases by collecting essential epidemiological and health economic data related to dystrophinopathy. The generated evidence can support DMD clinical trials and targeted drug development efforts while also aiding in diagnosis, treatment, scientific research, and policymaking concerning rare diseases in China.

This study builds upon the 2015 registry cohort (The CHFU database for dystrophinopathy) in East China, which has now expanded to encompass 2097 patients from 27 provinces across various regions of China. Through our diligent efforts, we have updated data regarding geographic distribution, age at diagnosis, phenotype and genotype correlations, family history, walking ability, cardiorespiratory function, clinical outcomes, the impact of corticosteroid treatment on motor function, and the usage of cardiac medication among patients with dystrophinopathy in China. Our objective is to provide high-quality medical care and treatment options to patients with dystrophinopathy through a reliable and current database. We anticipate that this database will support the research and development of new drugs for dystrophinopathy and offer real-world insights into the Chinese population.

## Materials and methods

### Institution and organization of the project

The CHFU database for dystrophinopathy is administered by Children’s Hospital of Fudan University. Neuromuscular Research Unit within the Department of Neurology oversees the development and management of the registry.

### Patients

The CHFU database for dystrophinopathy includes male Chinese patients who have been diagnosed with dystrophinopathy, a diagnosis that has been confirmed through genetic analysis or muscle biopsy (please refer to the Phenotypic classification section below).

### Method of registration and data collection

Information about the registry was conveyed to the male patients with dystrophinopathy attending the DMD Outpatient Clinic at our hospital. Patient data were included in the database with their informed consent, which detailed the database’s purpose and content, and registration was entirely voluntary. No personally identifiable information will be disclosed to any third party without explicit patient consent, and patients can request removal from the database immediately at any time. This study received approval from the Research Ethics Board of Children’s Hospital of Fudan University.

### Structure of the registry form and patients’ follow-up

The registry form’s design aligns with previous report [5]. The database undergoes semi-annual or annual updates through outpatient visits for ambulant patients and via telephone for non-ambulant patients who are unable to visit the outpatient clinic, particularly patients aged 18 and above.

### Diagnosis of dystrophinopathy

A diagnosis of dystrophinopathy is established in a proband exhibiting the characteristic clinical findings, elevated CK concentration, and/or the identification of a hemizygous pathogenic variant in the DMD gene through molecular genetic testing [33]. Patients who were diagnosed based on muscle biopsy results with dystrophin immunostaining were also included in our database. Multiplex ligation-dependent probe amplification (MLPA), a technique enabling the detection of large deletions and /or duplications in all 79 exons, has been developed and widely utilized [34]. In cases of single exon deletion, additional confirmation would be sought through PCR and sequencing. When MLPA failed to detect any variants, targeted sequencing or whole-exome sequencing would be conducted to screen for other types of variants. Alternatively, a muscle biopsy sample would be subjected to immunohistochemistry of tissue cryosections to determine the presence of dystrophin protein. Patients with no positive genetic results but with a confirmed dystrophinopathy diagnosis via muscle biopsy were recommended for RNA sequencing of the muscle tissue and long-read sequencing [35]. All identified variants were classified according to the American College of Medical Genetics and Genomics guidelines as benign, likely benign, uncertain significance, pathogenic, or likely pathogenic variants [36]. The numbering for DMD pathogenic variants was based on the cDNA sequence (reference transcript number: NM_004006.3).

### Phenotypic classification

Phenotypic classification is a collaborative effort between neurologists and genetic curators. It is primarily based on genetic analysis using the reading frame rule, while also taking into account clinical information and pathological data, including dystrophin immunostaining, when available. Four phenotypic subgroups have been defined, namely “DMD,” “BMD,” and “IMD” (Intermediate Muscular Dystrophy), and “pending” for patients whose phenotype cannot be determined due to factors such as age or incomplete clinical data [37].

### Loss of ambulation (LoA)

LoA is defined as the age reported by the participant or caregiver when continuous wheelchair use commences. This age is approximated to the nearest month and is verified by a trained clinical evaluator with the inability to perform the 10-meter run/walk assessment [38].

### Glucocorticoid (GC) treatment

During baseline and follow-up visits, we meticulously documented the timing of initiation or discontinuation, the specific drug, dosage, and administration pattern for both past and current GC regimens. Due to the limited number of DMD participants following intermittent regimens (e.g., 10-days-on/10-days-off, 10 days/month, and every other day), these regimens had been grouped together. The grouping criteria for Kaplan-Meier estimate were as follows: “untreated” (< 1 month’s use or never treated) and “treated” (≥ 1 year’s use, and starting ≥ 1 year before LoA), because a long-term effect cannot be attained with a short-term treatment [39].

### Statistical analysis

We performed a time-to-event analysis of each milestone with age (years) as time variable and patients reaching the milestone as event. Kaplan-Meier curves were employed to estimate the median age at each milestone for participant groups defined by GC treatment status while ambulant. The comparison of median age at each event was performed based on GC treatment status using the log-rank test, with statistical significance set at *p* < 0.05. All statistical analyses were performed with the SPSS software, version 25.

## Results

**Fig. 1:**
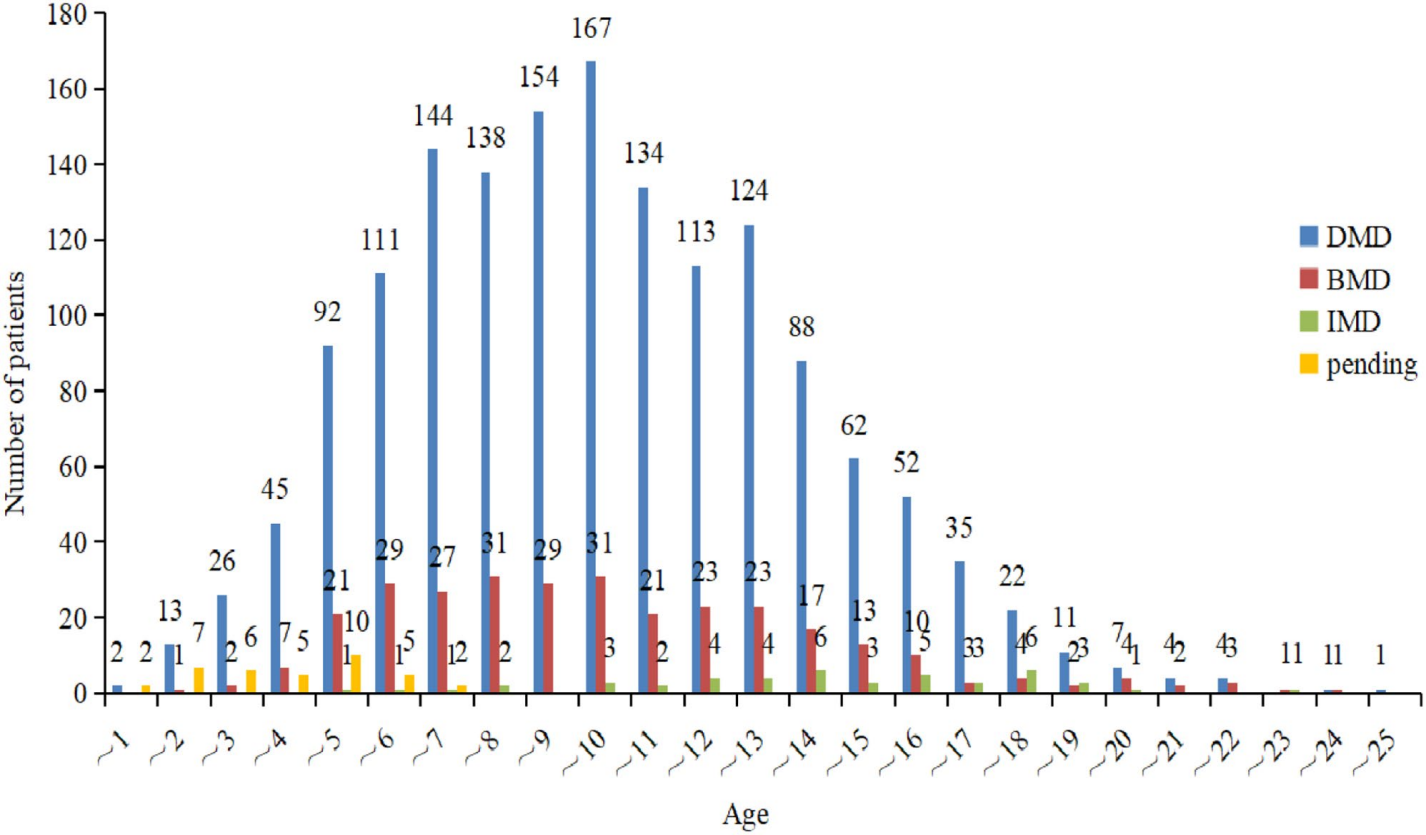
Ages of registered individuals. Most registrants were under 18 years of age

**Fig. 2:**
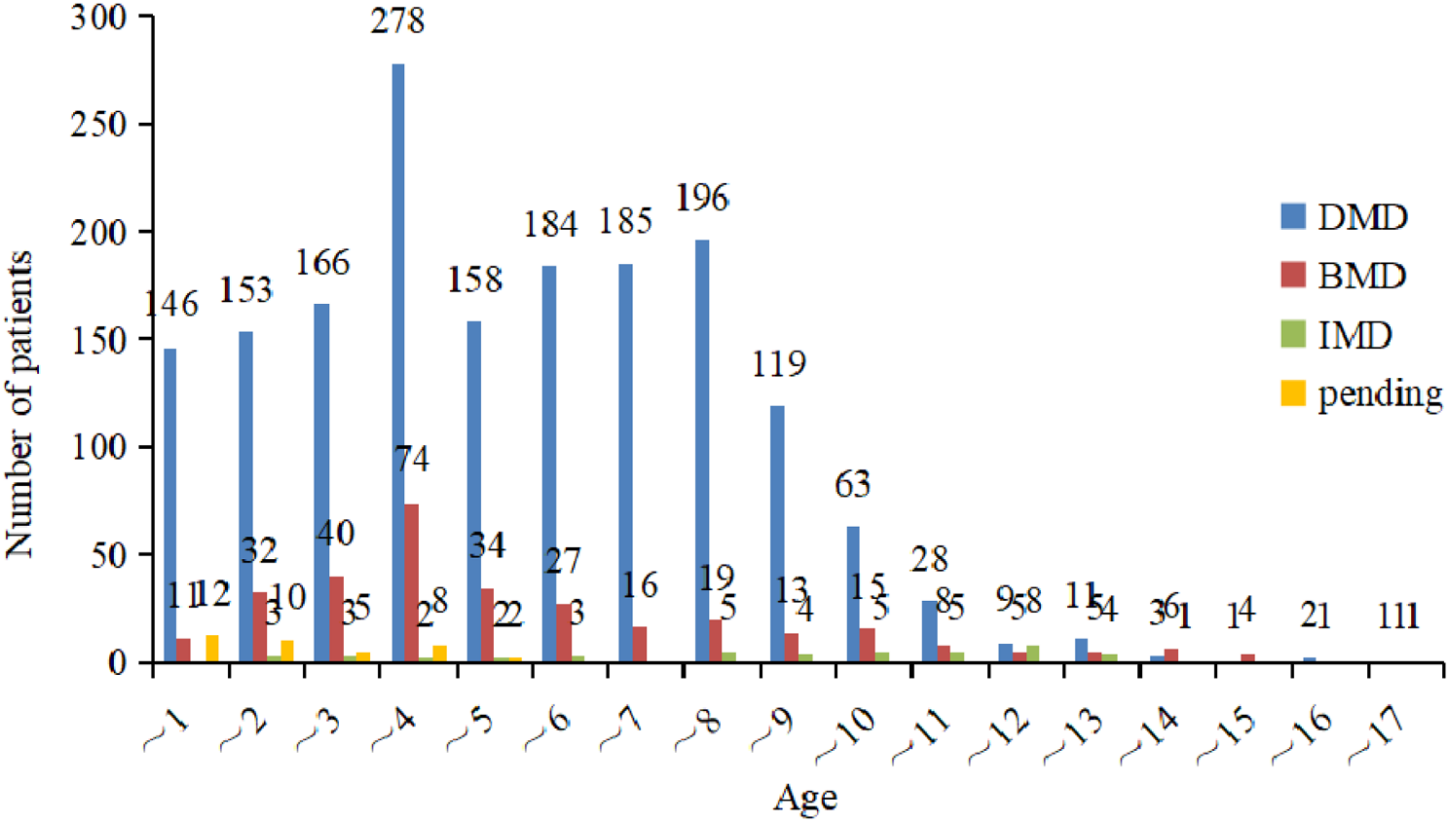
Age at the diagnosis. Most patients were diagnosed at 3–4 years old

As of August 2021, our database had a total of 2097 registered patients, categorized as follows: DMD (1703 cases), BMD (311 cases), IMD (46 cases), and individuals with an undetermined phenotype (“pending”) (37 cases). The majority of registrants were under 18 years of age. A total of 139 patients (6.6%) were lost to follow-up, and 20 patients (1%) passed away during the follow-up period. Genetic testing successfully identified a molecular diagnosis in 1949 boys. An additional 27 patients, whose genetic tests did not confirm dystrophinopathy, received a diagnosis through immunohistochemical staining of muscle biopsy specimens. Furthermore, 5.8% of patients without genetic testing were diagnosed via muscle biopsy. As of August 2021, the highest numbers of registrants within specific age groups were in the 9–10, 8–9, and 6–7-year-old categories (Fig. 1). Notably, a significant proportion of diagnoses occurred at ages 3–4, with 16.3% for DMD and 23.8% for BMD, primarily due to routine transaminase assessments conducted for kindergarten enrollment, a common practice in many Chinese provinces (Fig. 2).

**Fig. 3:**
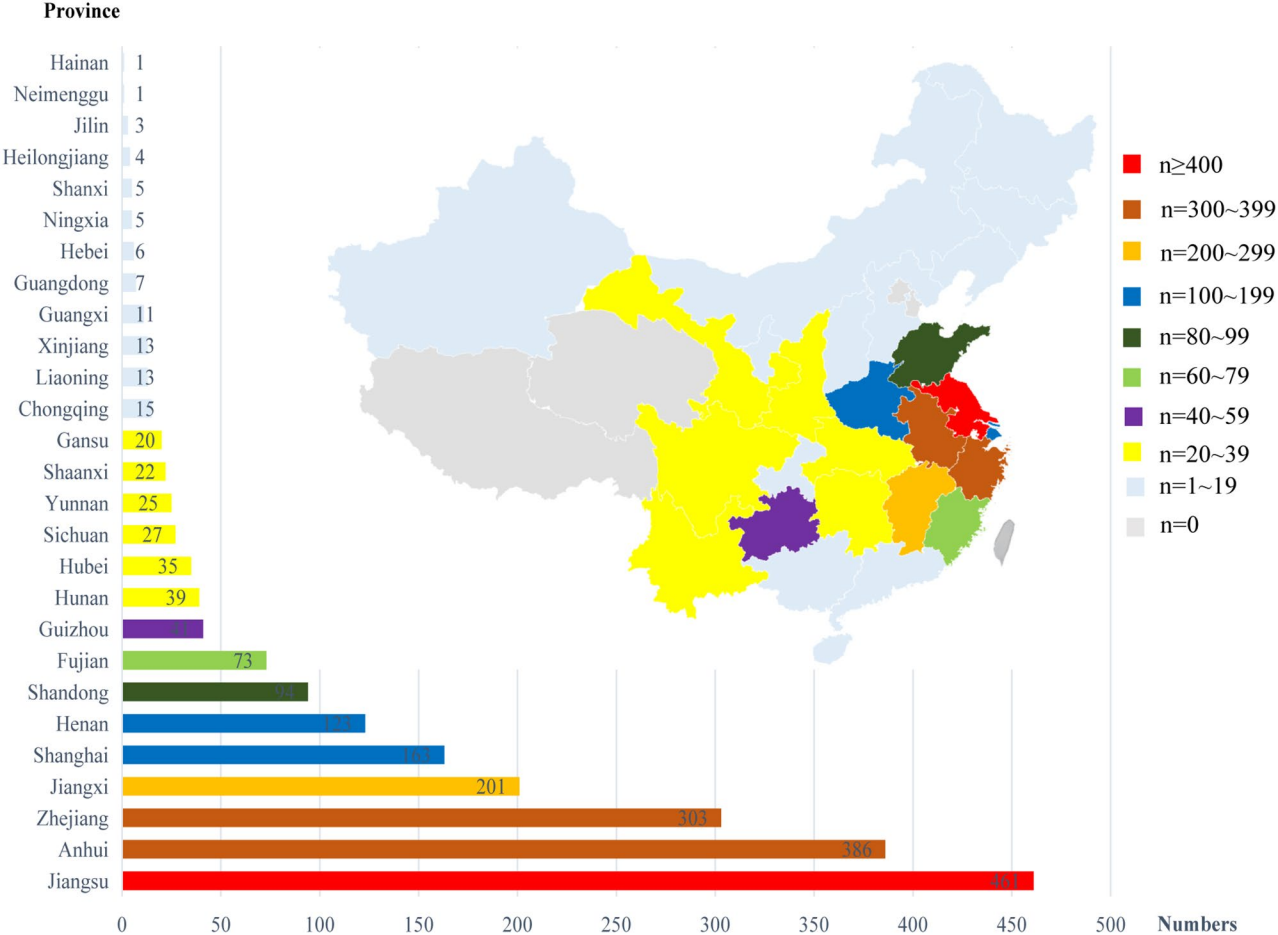
Geographical distribution of 2097 registrants

Patients with dystrophinopathy registered in our database hailed from 27 provinces across China, with the majority (80%) originating from East China, including Shanghai (163 cases) and surrounding provinces: Jiangsu (461), Anhui (386), Zhejiang (303), Jiangxi (201), Fujian (73), and Shandong (94). A smaller percentage of registrants resided in South Central China (199), Southwest China (106), Northwest China (61), Northeast China (19), South China (19), and North China (12) (Fig. 3).

Among the 1949 male patients in our database who underwent molecular diagnosis, the spectrum of identified variants included exonic deletions (66.6%), exonic duplications (10.7%), nonsense variants (10.3%), splice-site variants (4.5%), small deletions (3.5%), small insertions/duplications (1.8%), and missense variants (0.9%). Table 1 illustrated the distribution of variants in DMD/BMD patients. Exonic deletions were the most frequent, accounting for 65.7% in DMD and 78.5% in BMD cases, with the most frequent deletions occurring in exons 45–52. In DMD patients, the deletion of exons 45–50 (61 cases) was the most common deletion, while exon 2 duplication (17 cases) was the most common duplication, representing 5.8% (61/1052) of deletions and 9.6% (17/177) of duplications. In BMD patients, deletions of exons 45–47 (48 cases) and 45–48 (32 cases) accounted for 34.8% (80/230) of deletions. Table 2 displayed the most frequent patterns of large deletions and duplications in DMD and BMD patients. Among the 418 patients harboring small variants (Table S1&Table S2), 228 variants were previously reported, and 118 variants were unreported. However, the remaining 27 genetically undiagnosed patients were diagnosed based on muscle biopsy. Four deep intronic variants and two inversion variants were identified in seven patients (Table 3). A distinctive and rare causative variant located in intron 48, c.7098+1813G>T (reference transcript number: NM_004006.3), was pinpointed through skeletal muscle RNA analysis, where it was found to be hemizygous in two affected siblings (Patient 1&2). Additionally, the mother was identified as a carrier of this variant. This novel deep intronic variant resulted in the inclusion of a pseudoexon and a frameshift in coding sequences, leading to the truncation of dystrophin protein. The proband (Patient 1) began experiencing progressive muscle weakness at the age of 3 years. A clinical examination revealed pseudohypertrophy of the calf muscles, reduced tendon reflexes, winged scapula, lordosis, and Gower’s maneuver when the boy visited the outpatient clinic at 3.5 years of age. Despite initial negative results from MLPA analysis and next-generation sequencing, a subsequent muscle biopsy confirmed the diagnosis of DMD. Subsequently, the younger sibling (Patient 2) with a similar phenotype was also diagnosed with DMD. Patient 3 carried the c.9225–647A>G variant (reference transcript number: NM_004006.3), a pathogenic variant described in several studies [40–44]. This variant was inherited from his mother, and the patient exhibited an IMD phenotype. Although he displayed a positive Gower’s sign and a waddling gait, he retained the ability to jog at the age of 8 years and 7 months. Patient 4, a 14-year-old boy, was referred to our hospital due to gait abnormalities and progressive muscle weakness. He initially experienced gait abnormalities and muscle weakness at the age of seven years. Physical examination revealed calf muscle hypertrophy, a positive Gower’s sign, and a waddling gait. His serum creatine kinase (CK) levels were significantly elevated at 1400 U/L (normal: 0-164 U/L). Genetic testing and muscle biopsy were conducted at the age of 12.4 years because conventional genetic testing methods (MLPA analysis and next-generation sequencing) failed to identify the pathogenic variant in the DMD gene. Immunohistochemical tests on muscle biopsy displayed histological abnormalities in muscle tissue as well as faint and patchy dystrophin expression in muscle fibers. Despite initially negative results from mRNA-seq analysis of the muscle tissue, whole-genome sequencing was subsequently performed to identify an inversion variant of approximately 12,218 bp on chromosome Xp21, involving exon 5 of the DMD gene. This pathogenic variant was inherited from his mother. Patient 5, a 4.8-year-old boy, was referred to our hospital due to difficulty climbing stairs. Physical examination showed calf muscle hypertrophy and a positive Gower’s sign. His serum creatine kinase (CK) level was significantly elevated at 26,429 U/L (normal: 0-164 U/L). MLPA analysis and next-generation sequencing failed to identify any variants. However, a muscle biopsy confirmed the diagnosis of DMD. Subsequently, long-read sequencing was employed to identify the pathogenic variant. It revealed a definitive rare causative variant, c.9225–287C>A, located in intron 62, which had been previously reported in a Japanese patient [34]. The mother of Patient 5 was found to be a carrier. Patient 6, also referred to our hospital at the age of 4.8, experienced difficulty climbing stairs and frequent falls. Physical examination showed calf muscle hypertrophy and a positive Gower’s sign. The serum creatine kinase (CK) level was elevated to 10,256 U/L (normal: 0-164 U/L). MLPA analysis and next-generation sequencing failed to identify any variants. The diagnosis of DMD was confirmed based on a muscle biopsy. GC treatment (prednisone 0.75 mg/kg/d) was initiated at the age of five, and the patient consistently attended follow-up visits. At the age of 7.8, long-read sequencing was performed and identified the pathogenic variant c.3432+2240A>G in intron 25. The patient’s mother was identified as a carrier. Patient 7 was referred to our hospital at the age of 7.8 due to weakness in the lower legs, which began at the age of 4.3. Physical examination showed calf muscle hypertrophy, and a positive Gower’s sign. The serum creatine kinase (CK) level was elevated to 27,086 U/L (normal: 0-164 U/L). Despite negative results from MLPA analysis and next-generation sequencing, the confirmed diagnosis of DMD was based on the muscle biopsy findings. GC treatment (prednisone 0.75 mg/kg/d) was initiated at the age of 4.8, and regular follow-ups were conducted. At the age of 7.3, long-read sequencing was performed and revealed a de novo inversion variant of 296.507Kb on chromosome Xp21, which involved exon 2 to exon 5 of the DMD gene.

**Table 1:** Distribution of variants in DMD/BMD patients

Distribution of variants	DMD patients		BMD patients	
	No. of cases	% of cases	No. of cases	% of cases
Exonic deletion	1052	65.7%	230	78.5%
Exonic duplication	177	11.1%	22	7.5%
Exonic deletion and duplication	2	0.1%	0	0
Nonsense variants	186	11.6%	7	2.4%
Small insertion/duplication	34	2.1%	0	0
Small deletion	65	4.1%	3	1%
Small indel	1	0.1%	0	0
Splice-site variants	58	3.6%	20	6.8%
Missense variants	6	0.4%	7	2.4%
Synonymous variants	1	0.1%	0	0
No variants found*	19	1.2%	4	1.4%
	1601	100%	293	100%

*Diagnosis was made on the basis of absence of dystrophin expression and clinical manifestation

**Table 2:** The most frequent patterns of large deletions and duplications in DMD and BMD patients (reference transcript number: NM_004006.3)

DMD patients		BMD patients	
5’ hot spot	3’ hot spot	5’ hot spot	3’ hot spot
dup 2 (17)	del 45–50 (61)	del 3–4 (4)	del 45–47 (48)
dup 3–7 (12)	del 48–50 (53)	dup 2–7 (4)	del 45–48 (32)
dup 8–9 (11)	del 45–52 (51)		del 45–53 (20)
del 3–7 (8)	del 51 (48)		del 45–55 (19)
	del 45 (44)		del 48 (15)
	del 49–50 (41)		del 48–49 (14)
	del 46–47 (38)		
	del 44 (38)		
	del 49–52 (35)		
	del 48–52 (32)		
	del 46–48 (29)		
	del 52 (28)		
	del 46–52 (27)		
	del 46–51 (25)		
	del 45–54 (24)		
	del 46–50 (21)		
	del 50 (19)		
	del 48–54 (17)		
	del 51–53 (14)		
	del 50–52 (12)		
	del 51–55 (11)		
	del 53–55 (10)		

**Table 3:** Patients with ultra-rare variants (reference transcript number: NM_004006.3)

Patient number	Phenotype	Variant	Specific characteristic	Inheritance	Predicted ACMG classification
1&2	DMD	c.7098+1813G>T (Intron 48)	Deep intronic variant	Maternal	VUS
3	IMD	c.9225–647A>G (Intron 62)	Deep intronic variant	Maternal	Pathogenic
4	BMD	1036 bp intron 4 del, 12,218 bp inv, 608 bp delinsCTCAGTATC, intron 5 (ChrX:32,839,967–32,853,830)	Inversion	Maternal	Pathogenic
5	DMD	c.9225–287C>A (Intron 62)	Deep intronic variant	Maternal	VUS
6	DMD	c.3432+2240A>G (Intron 25)	Deep intronic variant	Maternal	Likely Pathogenic
7	DMD	ChrX:32,839,722–33,136,229	Inversion	De novo	Pathogenic

In addition, a notable percentage of DMD patients (55.3%) were eligible for exon-skipping therapy, with 12.9%, 10%, and 9.6% eligible for skipping exons 51, 53, and 45, respectively (Table 4).

**Table 4:** Candidates for exon skipping (exons 51, 53, and 45) to restore dystrophin expression (source: http://www.clinicaltrials.gov) (reference transcript number: NM_004006.3)

exon 51 skipping			exon 53 skipping			exon 45 skipping		
Deleted exon(s)	No. of individuals	% of registrants	Deleted exon(s)	No. of individuals	% of registrants	Deleted exon(s)	No. of individuals	% of registrants
19–50	1	0.1%	45–52	51	3.2%	12–44	1	0.1%
45–50	61	3.8%	47–52	3	0.2%	44	38	2.4%
47–50	4	0.2%	48–52	32	2%	46	2	0.1%
48–50	53	3.3%	49–52	35	2.2%	46–47	38	2.4%
49–50	41	2.6%	50–52	12	0.7%	46–48	29	1.8%
50	19	1.2%	52	28	1.7%	46–49	9	0.5%
52	28	1.7%				46–51	25	1.5%
						46–53	1	0.1%
						46–55	8	0.5%
						46–57	1	0.1%
						46–59	1	0.1%
						46–60	1	0.1%
total	207	12.9%		161	10%		154	9.6%

Some DMD boys (54.4%), IMD boys (39.1%), and a small percentage of BMD boys (1%) received glucocorticoid (GC) treatment. Among the 1550 DMD patients, 777 (50.1%) of follow-up patients were treated with GC. An additional 67 (4.3%) out of the 1550 DMD patients had previously used GC, with treatment lasting at least one month, but were not currently undergoing GC therapy. Additionally, 706 out of the 1550 (45.6%) DMD patients had never received GC treatment. Notably, 98.7% of DMD patients receiving GC therapy opted for daily prednisone (PRED), which was the most commonly prescribed regimen. Eleven DMD patients chose intermittent use, such as 10-days-on/10-days-off, 10 days/month, and every other day. Nineteen DMD patients switched from PRED to deflazacort (DFZ), and three later switched back to PRED. On average, the age at the initiation of GC treatment (excluding treatment started after the onset of LoA) was 6.3 ± 1.6 years (ranging from 1.8 to 12 years). The majority of DMD patients (26.8%) commenced GC treatment at 4–5 years of age. The mean duration of treatment while ambulant was 3 ± 1.7 years, with a range of 0.1–15.4 years, as detailed in Table 5.

**Table 5:** Glucocorticoid (GC) treatment of DMD patients while ambulant

	GC treatment	GC previously	GC naïve	Total
	n = 741	n = 67	n = 698	n = 1506
Age (years)	9.2 ± 2.2	12.5 ± 2.3	8.1 ± 3.4	8.8 ± 2.9
Age at diagnosis	4.9 ± 2.1	7.4 ± 1.6	4.6 ± 2.5	4.9 ± 2.4
Age at loss of ambulation	10.7 ± 1.6	10.5 ± 1.8	9.9 ± 1.3	10.2 ± 1.5
GC initiated	6.2 ± 1.5	7.7 ± 1.5	N/A	6.3 ± 1.6
GC duration	3.1 ± 1.7	2.7 ± 2	N/A	3 ± 1.7
GC stopped	N/A	10.1 ± 1.9	N/A	10.1 ± 1.9

Age and timing of milestones in years ± SD. N/A: not applicable. GC treatment: DMD patients used GC for at least one month and were currently undergoing GC therapy. GC previously: DMD patients had used GC for at least one month, but were not currently undergoing GC therapy. GC naïve: DMD patients used GC for less than one month or never used it.

In our database, 67.5% of DMD boys and 78.6% of IMD boys retained their ambulatory status, whereas all BMD boys were ambulant. Cardiac function was assessed in 49.8% of DMD boys, 63% of IMD boys, and 38.4% of BMD boys. Respiratory function assessments were conducted for only 26.6% of DMD boys, 45.7% of IMD boys, and 11.5% of BMD boys. Low cardiac function (ejection fraction [EF] < 55%) was detected in 0.6% of DMD patients, 4.3% of IMD patients, and 1% of BMD patients. A total of 7.4% of DMD patients, 8.3% of IMD patients, and 2.6% of BMD patients were prescribed at least one cardiac medication, as illustrated in Table 6. The average age at the initiation of cardiac medications was 10 ± 2 years (range, 3–17.6 years) for DMD patients, 13.2 ± 2.7 years (range, 9.3–17 years) for IMD patients, and 9.9 ± 3.4 years (range, 3.9–14.3 years) for BMD patients. Among the patients in our database, only four used ventilator support, and none underwent scoliosis surgery.

**Table 6:** Walking capability, medications, and intervention in the registrants with DMD and BMD

	DMD patients		BMD patients	
	No.of cases	% of cases	No.of cases	% of cases
**Walking capability**				
Not acquiring the ability to walk independently	7	0.5	0	0
Normal walking	1046	67.5%	305	100%
Not able to walk	497	32.1%	0	0
	1550	100%	305	100%
**Cardiac function**				
Normal	763	49.2%	114	37.4%
Dysfunction	9	0.6%	3	1%
Not performed	778	50.2%	188	61.6%
	1550	100%	305	100%
**Respiratory function**				
Normal	366	23.6%	35	11.5%
Dysfunction	47	3%	0	0
Not performed	1137	73.4%	270	88.5%
	1550	100%	305	100%
**Corticosteroid use**				
Current	777	50.1%	2	0.7%
Used to	67	4.3%	1	0.3%
Never	706	45.6%	302	99%
	1550	100%	305	100%
**Cardiac medication**				
Prescribed	114	7.4%	8	2.6%
Not prescribed	1436	92.6%	297	97.4%
	1550	100%	305	100%
**Drug**				
ACE-inhibitor	93	81.6%	6	75%
β-blocker	27	23.7%	5	62.5%
Diuretics	4	3.5%	3	37.5%
Other	6	5.3%	3	37.5%
	114*1		8*1	

*1. The number of registrants who were prescribed with cardiovascular medicines

## Discussion

Rare disease research is confronted with numerous challenges, including limited sample sizes, geographically dispersed patient populations, and inadequate follow-up, all of which hinder the acquisition of dependable epidemiological data. Large cohort studies and registries present a highly effective strategy for addressing the multifaceted obstacles inherent in researching rare diseases [45–47]. In August 2011, our hospital established a single-center dystrophinopathy registry database with the objective of investigating the characteristics of the Chinese dystrophinopathy population. The primary focus of this registry cohort is to delve into the intricate structural variations of the DMD gene, explore genotype/phenotype correlations, and assess the impacts of corticosteroid intervention. Additionally, this registry database serves as a vital platform for acquiring fundamental epidemiological data, evaluating health economic parameters, identifying potential drug targets, and facilitating clinical trials.

### Variant distribution, genotype/phenotype correlations, ultra-rare variants, family history and carrier screening

Between August 2011 and August 2021, a total of 2097 patients with dystrophinopathy were enrolled in the CHFU database for dystrophinopathy. Among these patients, 1976 underwent genetic testing, resulting in the identification of variants in 1949 patients. The most frequent variant type was exonic deletion, followed by exonic duplications, nonsense variants, splice-site variants, and small deletion variants. This pattern aligned with observations in other databases [37, 48] and previous reports within the Chinese population [49–51], albeit with some distinctions. Monaco et al. proposed the reading-frame rule as an explanation for the phenotypic differences between Duchenne and Becker patients [52]. In our database, 95.3% of DMD/BMD patients conformed to this rule. There are well-documented exceptions to the rule, such as exon 3–7 deletions [53], which were observed in eight DMD, one BMD, and six IMD patients, as well as in two patients with a pending phenotype. Additionally, we noted that exon 3–13 deletions (seven occurrences), exon 3–44 deletions/duplications (five/two occurrences), exon 3–30 deletions (five occurrences), and exon 3–18 deletions (three occurrences) were the most common exceptions to the reading frame rule. Annemieke Aartsma-Rus et al. suggested that deletions removing both the actin-binding domain and part of the central rod domain usually cause DMD, which may be explained by the fact that an additional actin-binding site is present in the central rod domain [53].

Ultra-rare variants were identified in seven patients. Among these, five patients had deep intronic variants, and two had inversion variants. For patients with variants that are difficult to determine the pathogenicity and show negative genomic testing but abnormal dystrophin, an analysis of muscle-derived RNA is an important diagnostic step [44]. Additionally, long-read sequencing and whole-genome sequencing may facilitate the identification of the ultra-rare variants in the DMD gene. Obtaining a definitive molecular diagnosis can effectively aid in providing accurate genetic counseling and diagnostic testing for family members.

Our data represent the largest cohort of male proband patients with independent variants reported in East China to date. This extensive genetic mapping of DMD allowed us to draw various correlations between DMD/BMD genotype landscapes and variant frequency, variant types, variant locations along the gene, and their effects on population genetic characteristics, as well as new potential personalized dystrophinopathy therapies. Furthermore, it’s worth noting that some patients with specific variants in our database could potentially benefit from novel genetic therapies for DMD. These therapies include stop codon readthrough therapy, which accounts for 11.6% of total variants and exon skipping therapy, which accounts for 84% of deletions and 55.3% of total variants. The top 10 exon skips that could be applicable to the largest group of patients were skipping of exon 51 (12.9% of total variants), 53 (10%), 45 (9.6%), 44 (5.7%), 50 (4.8%), 52 (4.4%), 55 (3.9%), 43 (3.2%), and 8 (0.7%).

In our study, we found that 13% of the probands had a positive family history of the disease. Additionally, the carrier status of 1242 probands’ mothers was investigated, with 799 mothers carrying the same variant (64.3%). This was consistent with previous reports from the European and Chinese populations with dystrophinopathy [37, 49, 50]. Furthermore, we identified five cases with mosaicism in the mothers through the prenatal diagnosis of the fetus or the probands’ siblings, contributing to our understanding of the genetic characteristics of this condition.

### Glucocorticoid treatment

Glucocorticoid treatment is well-known to be associated with reduced risk of losing clinically meaningful mobility and upper limb disease progression milestones across the lifespan as well as reduced risk of death for patients with DMD [17]. The usage of GC therapy has notably risen to 54.4%, in contrast to our prior study [5] and findings in various regions of China [30]. Nonetheless, in comparison to certain developed nations [54], the use of glucocorticoids remains relatively low. The primary reasons for not receiving glucocorticoid therapy included parental rejection due to potential side effects like weight gain and increased osteoporosis risk and their understanding that GC treatment could only provide limited improvement rather than a cure for the disease. Additionally, some families with limited financial means couldn’t afford glucocorticoid therapy.

**Fig. 4:**
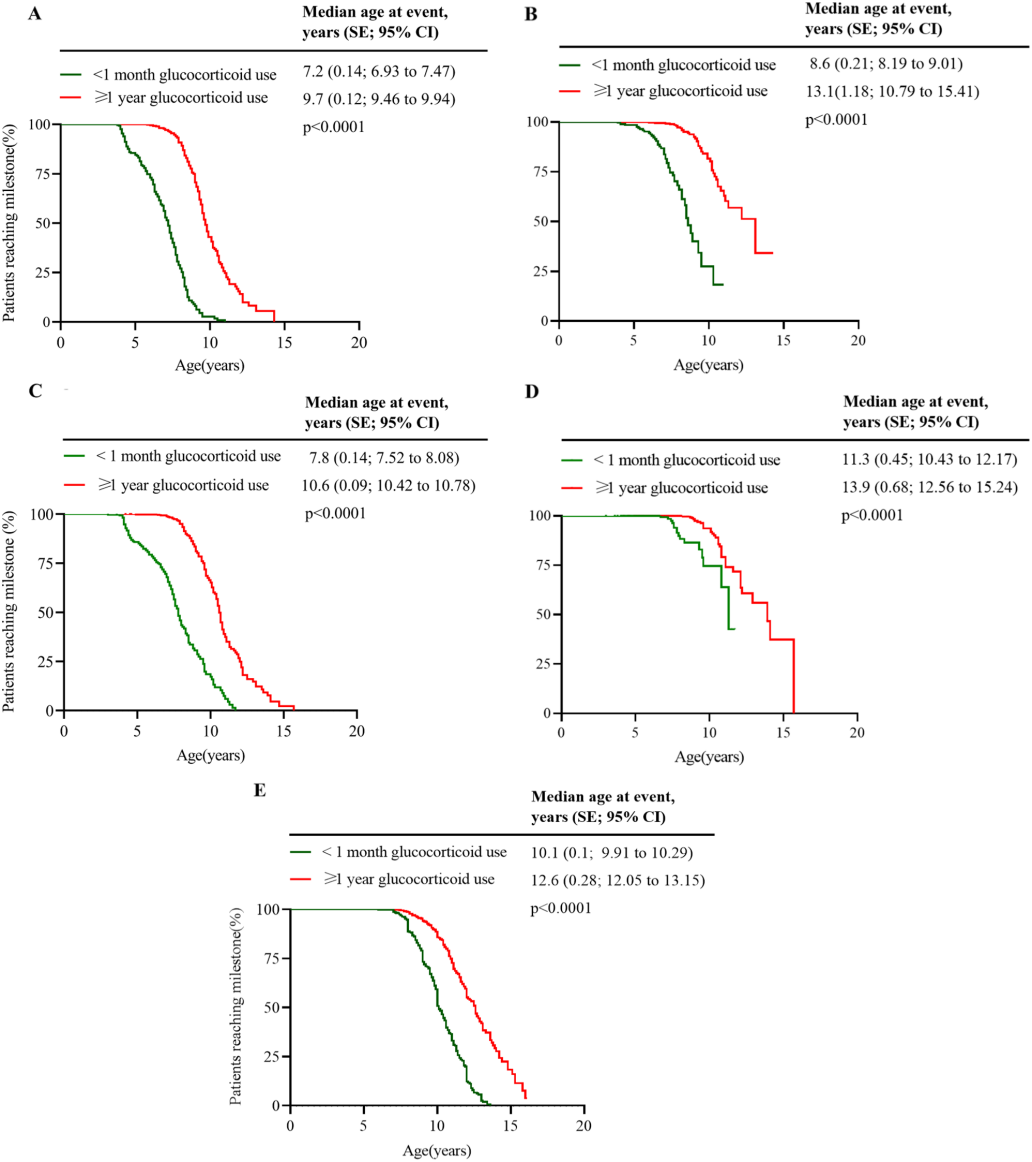
Time to ambulatory milestones. Kaplan-Meier analyses comparing cumulative glucocorticoid use (< 1 month or never treated vs. ≥ 1 year) for (**A**) age at transition to stand from supine of 5 s or greater; (**B**) age at transition to stand from supine of 10 s or greater; (**C**) age at transition to walk/run 10 m of 6 s or greater; (**D**) age at transition to walk/run 10 m of 12 s or greater; and (**E**) age at loss of ambulation (unable to ambulate 10 m)

Kaplan-Meier analysis in our study showed that DMD patients who received GC treatment for at least one year while still ambulatory experienced a median age at LoA that was 2.5 years later compared to those who remained untreated or were treated for less than one month (12.6 years vs. 10.1 years, *n* = 665 vs. 688, log-rank *p* < 0.0001) (see Fig. 4). Numerous studies on DMD have emphasized the time to LoA as their primary outcome measure. For instance, King et al. observed that DMD males who received corticosteroid treatment for a minimum of one year (*n* = 75) walked for an additional 3.3 years compared to untreated males (*n* = 68) [55]. Kim et al. noted that long-term corticosteroid treatment at an earlier stage delayed time to loss of ambulation with cases in the long-term treatment group stopping walk 2 years later, at an average age of 12.3 years [56]. The CINRG Duchenne Natural History Study discovered that receiving GC treatment for one year or longer extended the median age at LoA by 3.4 years, when compared to those treated for less than one month or never treated at all (13.4 years vs. 10 years, *n* = 330 vs. 73, log-rank *p* < 0.0001) [17]. More recently, a study involving 1163 Chinese DMD patients indicated significant positive responses to GC treatment. The median ages at LoA for GC-naïve, prednisone/prednisolone-treated, and deflazacort-treated groups were 10.23, 12.02, and 13.95 years, respectively [31]. We also compared patients treated with GC versus those who were GC-naïve patients in terms of the age at which they transitioned to being able to stand for ≥ 5 s and ≥ 10 s from a supine position, which tended to be more delayed in GC-treated patients than in GC-naïve patients. Additionally, the age at which they transitioned to walking or running for ≥ 6 s and ≥ 12 s in a 10-meter walk/run test also tended to be more delayed in GC-treated patients than in those who were GC-naïve (see Fig. 4). Clearly, GC treatment exerted a pronounced protective effect on the decline in motor function in Chinese patients with DMD. However, we observed that due to rapid weight gain, some patients may not derive the same benefits from GC treatment in terms of delaying the age of LoA, and some chose to discontinue the treatment. Nevertheless, the situation may improve in the near future, as a New Drug Application filing for vamorolone, a first-in-class steroid medication that targets the same receptors as corticosteroids but with fewer side effects, has been submitted to the FDA [26].

### Cardiac and pulmonary management

In the realm of DMD patient care, cardiac and pulmonary management holds paramount importance. The objective here is to enhance the quality of life by actively monitoring and addressing heart and lung complications, aiming to delay their onset and progression. Recognizing that adverse myocardial changes occur before noticeable cardiac issues and at a younger age than previously thought, as outlined in the 2018 DMD Care Guidelines, it is now recommended to commence annual cardiac screenings upon diagnosis [57]. The initial cardiac evaluation should encompass a thorough review of the patient’s medical and family cardiac history, along with a physical examination, electrocardiogram (ECG), and non-invasive imaging utilizing either an echocardiogram or cardiac MRI (CMRI), depending on the child’s age and ability to cooperate. For patients with established cardiac abnormalities, more frequent visits may be necessary [57, 58]. Based on our dataset, we have observed that ECG can identify abnormalities at a very early age, preceding other cardiac function tests in DMD patients. Notable ECG changes include heightened left ventricular activity, sinus arrhythmia, shortened PR interval, T wave alterations, left and right ventricular hypertrophy, prolonged corrected QT interval, and Q waves. In our study, a significant portion (53%) of DMD patients who underwent ECG examination before the age of six displayed these abnormalities well before the onset of clinical symptoms. Similarly, ECG abnormalities were commonly found in patients with BMD, although they were generally nonspecific, in line with prior reports [59].

As there are no targeted cardiac therapies specific to dystrophinopathy, traditional heart failure treatment approaches are employed. Angiotensin-converting enzyme (ACE) inhibitors or angiotensin receptor blockers are commonly used as the initial treatment options for heart-related issues in DMD [58]. In our clinical practice, cardiologists usually initiate ACE inhibitor use in asymptomatic boys approaching the age of 10 with normal left ventricular systolic function, following discussions with the family about potential benefits and risks. Regardless of age, pharmacological treatment should commence when heart failure symptoms emerge or abnormalities are detected in imaging studies (ECG, CMR, or echocardiogram). Our database indicates that 7.4% of DMD, 8.3% of IMD, and 2.6% of BMD registrants were prescribed at least one cardiac medication, with ACE inhibitors being the most commonly prescribed, as indicated in Table 6. Although the use of cardiac medications among DMD patients is lower than that of developed countries [20], there has been a significant increase compared to our previous study (2.1%) [5]. This increase can be attributed to the growing adoption of the 2018 DMD Care Guidelines [57], which has heightened awareness among patients’ families regarding the importance of cardiac care and improved the understanding of cardiac medication management among specialists. It is anticipated that a standardized approach to cardiac medication management for DMD will become more widespread in China in the near future.

Minimal pulmonary function assessment, such as measuring forced vital capacity (at least once a year), helps the child to become familiar with the equipment and allows the medical team to determine the maximum respiratory function while the boy with DMD can still walk independently. The primary emphasis on pulmonary assessment occurs after the loss of independent walking [57]. However, pulmonary management remains suboptimal in our patient group. Only 26.6% of DMD patients underwent pulmonary function testing, primarily due to cognitive impairments that made reliable testing difficult. In our database, abnormalities were detected in only 3% of DMD patients, mostly due to poor compliance with follow-up, especially among wheelchair users. Ventilatory assistance significantly improves the survival of DMD patients, and the median survival age in these patients now reaches 40, without a decrease in their quality of life [60]. However, only four patients in our database used ventilator support. Despite the passage of time, little has changed in the care of these patients due to insufficient awareness among physicians and families about the importance of these measures for the patient’s quality of life, as well as a lack of medical insurance coverage for treatment expenses. With the increasing use of GC treatment and the approval of new drugs in recent years, it is crucial to raise awareness about the disease among the general public, physicians, and government organizations.

### Cause of death in DMD children

During the follow-up period, a total of twenty patients passed away, with seventeen of them succumbing to complications related to DMD. The mean age at death due to DMD was 14.4 ± 2.2 years, ranging from 10.8 to 20.9 years, except for two patients who did not die from DMD complications. One patient died of pneumonia at the age of three, while the other succumbed to acute leukemia at the age of seven. The exact cause of death for one patient remained unclear.

## Conclusions

The CHFU database for dystrophinopathy has played a vital role in providing clinicians with an essential tool for selecting patients based on predetermined genetic and phenotypic criteria for clinical trials of new drugs for dystrophinopathy. The registration of 2097 patients in the database is significant, as it enables the acquisition of accurate knowledge regarding the natural history of patients with dystrophinopathy in China. This knowledge provides clinicians with insights into the effectiveness of therapeutic interventions and facilitates the development of practical and cost-effective strategies for managing patients with dystrophinopathy.

### Electronic supplementary material

Below is the link to the electronic supplementary material.

Supplementary Material 1

Supplementary Material 2

## Data Availability

The data that support the findings of this study are not openly available due to reasons of sensitivity and are available from the corresponding author upon reasonable request. Data are located in controlled access data storage at Children’s Hospital of Fudan University.

## Abbreviations

DMD: Duchenne muscular dystrophy
IMD: Intermediate muscular dystrophy
BMD: Becker muscular dystrophy
CK: Creatine Kinase
MLPA: Multiplex Ligation-dependent Probe Amplification
LoA: Loss of ambulation
GC: Glucocorticoid
DFZ: Deflazacort
PRED: Prednisone or prednisolone
