# Supplementary material for: Comprehensive analysis of 2097 patients with dystrophinopathy based on a database from 2011 to 2021
Source: Orphanet J Rare Dis. 2024 Aug 24;19:311. doi: 10.1186/s13023-024-03217-7 (PMC11344408; doi:10.1186/s13023-024-03217-7)
Supplement: Supplementary file 1 — Supplementary Material 1 [file 13023_2024_3217_MOESM1_ESM.docx]

Table S1. Clinical and dystrophin immunostaining data of patients with a small sequence variant yet unpublished in the literature/databases

| Patient | Phenotype | Age  (y) | Family history | ambulatory | | Age (y)  at not  ambulatory | Dystrophin immunostaining | Exon | Nucleotide change  (NM_004006.3) | Predicted amino acid  change | Predicted consequence | Predicted ACMG classification |
| --- | --- | --- | --- | --- | --- | --- | --- | --- | --- | --- | --- | --- |
| 1 | DMD | 14.4 | No | No | 12.3 | | negative | 55 | c.8147_8148insCAGAAGCTGAAACAACTGCCAATGTCCTACA | p.(Gln2716Hisfs*4) | Frameshift | LP |
| 2 | DMD | 13.1 | Yes | No | 12.2 | | negative | 45 | c.6540del | p.(Leu2181Tyrfs*8) | Frameshift | LP |
| 3 | DMD | 9.3 | No | Yes |  | | negative | 34 | c.4711_4712insTG | p.(Lys1571Metfs*11) | Frameshift | LP |
| 4 | DMD | 12.4 | No | No | 10.6 | | negative | 24 | c.3211_3212insTGATGTTTTT | p.(Lys1072Metfs*29) | Frameshift | LP |
| 5 | DMD | 16.3 | No | No | 12.2 | | negative | 75 | c.10654dup | p.(Ser3552Lysfs*6) | Frameshift | LP |
| 6 | DMD | 14.7 | No | No | 11.2 | | negative | 26 | c.3532_3533dup | p.(Glu1179Lysfs*23) | Frameshift | LP |
| 7 | DMD | 16.3 | No | No | 15.8 | | negative | 23 | c.3054T>A | p.(Tyr1018*) | Nonsense | LP |
| 8 | DMD | 10.2 | No | Yes |  | | negative | 26 | c.3583A>T | p.(Lys1195*) | Nonsense | LP |
| 9 | DMD | 13.9 | No | No | 10.3 | | not done | int17 | c.2168+2_2168+3del |  | Splice-site | VUS |
| 10 | DMD | 12.1 | No | No | 8.5 | | negative | 7 | c.613dup | p.(Tyr205Leufs*12) | Frameshift | LP |
| 11 | DMD | 8.7 | No | Yes |  | | negative | 20 | c.2510del | p.(Asn837Ilefs*9) | Frameshift | LP |
| 12 | DMD | 9.3 | No | Yes |  | | negative | 29 | c.4053G>A | p.(Trp1351*) | Nonsense | LP |
| 13 | DMD | 10.2 | No | Yes |  | | negative | 26 | c.3540T>A | p.(Tyr1180*) | Nonsense | LP |
| 14 | DMD | 6.5 | No | Yes |  | | negative | 15 | c.1793C>A | p.(Ser598*) | Nonsense | LP |
| 15 | DMD | 12.7 | No | Yes |  | | negative | 66 | c.9634G>T | p.(Glu3212*) | Nonsense | LP |
| 16 | DMD | 8.8 | Yes | Yes |  | | not done | 18 | c.2280del | p.(Glu761Lysfs*6) | Frameshift | LP |
| 17 | DMD | 13.4 | Yes | No | 9.0 | | not done | 38 | c.5403_5418del | p.(Gln1802*) | Nonsense | LP |
| 18 | DMD | 13.4 | Yes | No | 11.4 | | not done | 38 | c.5403_5418del | p.(Gln1802*) | Nonsense | LP |
| 19 | DMD | 13.6 | Yes | No | 8.5 | | negative | 8 | c.803T>A | p.(Leu268*) | Nonsense | LP |
| 20 | DMD | 8.4 | No | Yes |  | | not done | 61 | c.9132del | p.(Phe3045Leufs*44) | Frameshift | LP |
| 21 | BMD | 10.8 | Yes | Yes |  | | not done | int25 | c.3433-1G>C |  | Splice-site | VUS |
| 22 | DMD | 17.9 | No | No | 10.0 | | not done | int54 | c.8028-1G>T |  | Splice-site | VUS |
| 23 | DMD | 7.3 | No | Yes |  | | negative | 26 | c.3601A>T | p.(Lys1201*) | Nonsense | LP |
| 24 | DMD | 5.1 | No | Yes |  | | negative | 46 | c.6643G>T | p.(Glu2215*) | Nonsense | LP |
| 25 | DMD | 10.7 | No | Yes |  | | negative | 20 | c.2621_2622+40del |  | Splice-site | VUS |
| 26 | DMD | 6.8 | No | Yes |  | | not done | 32 | c.4501_4502del | p.(Gln1501Alafs*9) | Frameshift | LP |
| 27 | DMD | 7.2 | No | Yes |  | | negative | Int53 | c.7873-11A>G |  | Splice-site | VUS |
| 28 | DMD | 5.9 | No | Yes |  | | negative | 12 | c.1478_1479dup | p.(Lys494Ilefs*8) | Frameshift | LP |
| 29 | DMD | 13.8 | No | No | 9.8 | | not done | 7 | c.549del | p.(Trp183*) | Nonsense | LP |
| 30 | DMD | 10.6 | No | Yes |  | | not done | 10 | c.982A>T | p.(Lys328*) | Nonsense | LP |
| 31 | BMD | 9.6 | Unknown | Yes |  | | faint | int46 | c.6763-1G>A |  | Splice-site | VUS |
| 32 | DMD | 9.5 | No | Yes |  | | not done | 17 | c.2155G>T | p.(Glu719*) | Nonsense | LP |
| 33 | DMD | 8.0 | No | Yes |  | | not done | 14 | c.1700_1701insC | p.(Glu567Aspfs*6) | Frameshift | LP |
| 34 | DMD | 9.8 | No | Yes |  | | not done | 55 | c.8189del | p.(Gly2730Glufs*2) | Frameshift | LP |
| 35 | DMD | 8.7 | No | Yes |  | | not done | 13 | c.1590_1591del | p.(Glu531Thrfs*3) | Frameshift | LP |
| 36 | IMD | 7.1 | No | Yes |  | | faint&patchy | 22 | c.2839A>C | p.(Thr947Pro) | Missense | VUS |
| 37 | DMD | 11.5 | Unknown | No | 8.7 | | not done | 23 | c.3042_3052dup | p.(Tyr1018Leufs*30) | Frameshift | LP |
| 38 | DMD | 6.8 | No | Yes |  | | negative | 35 | c.5012T>G | p.(Leu1671*) | Nonsense | LP |
| 39 | DMD | 9.7 | No | Yes |  | | negative | int25 | c.3433-1G>T |  | Splice-site | VUS |
| 40 | DMD | 12.6 | No | No | 9.2 | | not done | 17 | c.2013del | p.(Thr672Leufs*5) | Frameshift | LP |
| 41 | DMD | 5.9 | Yes | Yes |  | | not done | 20 | c.2620A>T | p.(Lys874*) | Nonsense | LP |
| 42 | BMD | 10.0 | No | Yes |  | | not done | int7 | c.650-2A>T |  | Splice-site | VUS |
| 43 | DMD | 5.9 | No | Yes |  | | not done | 30 | c.4109_4110insTCCA | p.(Gln1370Hisfs*8) | Frameshift | LP |
| 44 | BMD | 13.6 | No | Yes |  | | faint&patchy | int41 | c.5922+1G>A |  | Splice-site | VUS |
| 45 | DMD | 7.2 | No | Yes |  | | not done | 67 | c.9711dup | p.(Leu3238Serfs*3) | Frameshift | LP |
| 46 | DMD | 10.8 | No | No | 10.0 | | not done | 11 | c.1166_1185del | p.(Leu389Cysfs*3) | Frameshift | LP |
| 47 | BMD | 13.9 | No | Yes |  | | faint&patchy | 6 | c.510T>A | p.(Asn170Lys) | Missense | VUS |
| 48 | DMD | 5.7 | No | Yes |  | | not done | 75 | c.10681G>T | p.(Glu3561*) | Nonsense | LP |
| 49 | DMD | 4.3 | No | Yes |  | | not done | 53 | c.7745C>G | p.(Ser2582*) | Nonsense | P |
| 50 | DMD | 8.1 | No | Yes |  | | not done | 57 | c.8471_8472del | p.(Lys2824Argfs*2) | Frameshift | LP |
| 51 | DMD | 7.5 | No | Yes |  | | negative | 32 | c.4350_4351insA | p.(Leu1451Ilefs*18) | Frameshift | LP |
| 52 | IMD | 15.9 | No | Yes |  | | negative | 8 | c.826_831+1delinsTCTA |  | Splice-site | VUS |
| 53 | DMD | 12.1 | No | No | 8.1 | | not done | 67 | c.9767del | p.(Gly3256Alafs*27) | Frameshift | LP |
| 54 | DMD | 12.9 | No | Yes |  | | negative | 35 | c.4977C>G | p.(Asn1659Lys） | Missense | VUS |
| 55 | DMD | 9.6 | No | Yes |  | | not done | 47 | c.6806_6809del | p.(Leu2270Metfs*9) | Frameshift | LP |
| 56 | DMD | 9.8 | No | Yes |  | | negative | 23 | c.3057_3060del | p.(Ser1020Asnfs*23) | Frameshift | LP |
| 57 | DMD | 5.4 | No | Yes |  | | not done | 8 | c.717dup | p.(Pro240Alafs*7) | Frameshift | LP |
| 58 | DMD | 10.6 | No | No | 10.1 | | not done | 38 | c.5446_5447del | p.(Met1816Glufs*2) | Frameshift | LP |
| 59 | BMD | 14.1 | No | Yes |  | | faint&patchy | 36 | c.5153del | p.(Lys1718Serfs*3) | Frameshift | LP |
| 60 | DMD | 9.4 | No | Yes |  | | not done | 21 | c.2656_2659del | p.(Gln886Leufs*4) | Frameshift | LP |
| 61 | BMD | 5.8 | No | Yes |  | | faint&patchy | 6 | c.496G>C | p.(Gly166Arg) | Missense | VUS |
| 62 | DMD | 4.6 | No | Yes |  | | not done | 38 | c.5374A>T | p.(Lys1792*) | Nonsense | LP |
| 63 | DMD | 6.9 | Yes | Yes |  | | not done | 8 | c.717dup | p.(Pro240Alafs*7) | Frameshift | LP |
| 64 | DMD | 6.1 | No | Yes |  | | not done | 67 | c.9777dup | p.(Glu3260*) | Nonsense | LP |
| 65 | DMD | 5.5 | No | Yes |  | | not done | 46 | c.6719del | p.(Pro2240Leufs*7) | Frameshift | LP |
| 66 | pending | 4.1 | Yes | Yes |  | | not done | 41 | c.5740G>T | p.(Glu1914*) | Nonsense | LP |
| 67 | DMD | 5.3 | No | Yes |  | | not done | 8 | c.804dup | p.(His269Thrfs*19) | Frameshift | LP |
| 68 | DMD | 9.9 | No | Yes |  | | not done | 57 | c.8524C>T | p.(Gln2842*) | Nonsense | P |
| 69 | DMD | 5.1 | No | Yes |  | | not done | 21 | c.2782A>T | p.(Arg928*) | Nonsense | LP |
| 70 | DMD | 9.1 | No | Yes |  | | not done | 23 | c.3070G>T | p.(Glu1024*) | Nonsense | LP |
| 71 | DMD | 4.5 | Unknown | Yes |  | | not done | 16 | c.1969A>T | p.(Lys657*) | Nonsense | LP |
| 72 | DMD | 5.1 | No | Yes |  | | negative | 33 | c.4552del | p.(Glu1518Lysfs*5) | Frameshift | LP |
| 73 | BMD | 7.0 | No | Yes |  | | faint&patchy | 4 | c.251T>G | p.(Leu84Trp) | Missense | VUS |
| 74 | DMD | 8.2 | No | Yes |  | | not done | 47 | c.6823G>T | p.(Gly2275*) | Nonsense | LP |
| 75 | DMD | 4.4 | No | Yes |  | | not done | 56 | c.8379del | p.(Leu2794Serfs*30) | Frameshift | LP |
| 76 | DMD | 4.9 | No | Yes |  | | negative | 39 | c.5544_5569del | p.(Ile1849Thrfs*2) | Frameshift | LP |
| 77 | DMD | 5.0 | No | Yes |  | | not done | 70 | c.10123del | p.(Val3375Tyrfs*2) | Frameshift | LP |
| 78 | DMD | 4.4 | No | Yes |  | | not done | 48 | c.6982A>T | p.(Lys2328*) | Nonsense | LP |
| 79 | pending | 4.1 | No | Yes |  | | not done | int24 | c.3276+1_3276+6del |  | Splice-site | VUS |
| 80 | DMD | 5.0 | No | Yes |  | | not done | 65 | c.9519_9520del | p.(Cys3174Argfs*13) | Frameshift | LP |
| 81 | DMD | 4.8 | No | Yes |  | | negative | 32 | c.4474_4475del | p.(Ser1492Cysfs*18) | Frameshift | LP |
| 82 | DMD | 4.4 | Yes | Yes |  | | negative | 51 | c.7542G>T | p.(Lys2514Asn) | Missense | VUS |
| 83 | DMD | 5.2 | No | Yes |  | | negative | int33 | c.4675-2A>T |  | Splice-site | VUS |
| 84 | DMD | 7.1 | No | Yes |  | | negative | 53 | c.7696dup | p.(Glu2566Glyfs*12) | Frameshift | LP |
| 85 | DMD | 4.3 | No | Yes |  | | not done | 39 | c.5503del | p.(Gln1835Asnfs*13) | Frameshift | LP |
| 86 | DMD | 6.9 | No | Yes |  | | not done | 53 | c.7834dup | p.(Thr2612Asnfs*31) | Frameshift | LP |
| 87 | DMD | 5.2 | No | Yes |  | | not done | 70 | c.10211del | p.(Asp3404Alafs*9) | Frameshift | LP |
| 88 | DMD | 4.0 | No | Yes |  | | not done | 29 | c.4047del | p.(Arg1350Valfs*10) | Frameshift | LP |
| 89 | DMD | 5.5 | No | Yes |  | | not done | 7 | c.589dup | p.(Glu197Glyfs*20) | Frameshift | LP |
| 90 | DMD | 4.9 | No | Yes |  | | negative | 41 | c.5915_5922+19delinsTATG | p.(Ala1972Valfs*10) | Frameshift | LP |
| 91 | DMD | 7.7 | Yes | Yes |  | | not done | 35 | c.4973del | p.(Ser1658Ilefs*4) | Frameshift | LP |
| 92 | DMD | 6.0 | Yes | Yes |  | | not done | 35 | c.4973del | p.(Ser1658Ilefs*4) | Frameshift | LP |
| 93 | DMD | 5.7 | Yes | Yes |  | | not done | int62 | c.9225-1G>C |  | Splice-site | VUS |
| 94 | DMD | 6.6 | No | Yes |  | | not done | 4 | c.234dup | p.(Lys79Glnfs*10) | Frameshift | LP |
| 95 | DMD | 4.5 | No | Yes |  | | negative | int5 | c.358-13T>G |  | Splice-site | VUS |
| 96 | DMD | 3.7 | No | Yes |  | | not done | 54 | c.7895del | p.(Gln2632Argfs*6) | Frameshift | LP |
| 97 | BMD | 10.5 | No | Yes |  | | not done | int39 | c.5586+1G>T |  | Splice-site | VUS |
| 98 | pending | 1.5 | No | Yes |  | | not done | 64 | c.9316_9319dup | p.(Arg3107Ilefs*2) | Frameshift | LP |
| 99 | DMD | 10.5 | No | No | 10.3 | | not done | 33 | c.4542del | p.(Val1515*) | Nonsense | LP |
| 100 | DMD | 7.7 | No | Yes |  | | not done | 13 | c.1499del | p.(Leu500Glnfs*17) | Frameshift | LP |
| 101 | DMD | 4.6 | No | Yes |  | | not done | 66 | c.9624dup | p.(Ala3209Serfs*22) | Frameshift | LP |
| 102 | pending | 3.1 | No | Yes |  | | not done | 19 | c.2326A>T | p.(Lys776*) | Nonsense | LP |
| 103 | DMD | 4.0 | No | Yes |  | | not done | 65 | c.9455del | p.(Asn3152Ilefs*3) | Frameshift | LP |
| 104 | pending | 2.4 | No | Yes |  | | faint&patchy | 65 | c.9527A>G | p.(Asp3176Gly) | Missense | VUS |
| 105 | DMD | 5.0 | No | Yes |  | | not done | 30 | c.4096G>T | p.(Glu1366*) | Nonsense | LP |
| 106 | BMD | 4.0 | No | Yes |  | | faint&patchy | 34 | c.4845G>C | p.(Lys1615Asn) | Missense | VUS |
| 107 | BMD | 13 | No | Yes |  | | faint&patchy | 7 | c.584G>C | p.(Arg195Pro) | Missense | VUS |
| 108 | pending | 3.0 | No | Yes |  | | not done | int54 | c.8028-1G>A |  | Splice-site | VUS |
| 109 | pending | 2.8 | No | Yes |  | | not done | 37 | c.5198_5199del | p.(Ser1733Tyrfs*3) | Frameshift | LP |
| 110 | DMD | 12.4 | No | No | 10.8 | | negative | 36 | c.5056C>T | p.(Gln1686*) | Nonsense | LP |
| 111 | DMD | 7.0 | Yes | Yes |  | | not done | 57 | c.8446G>T | p.(Glu2816*) | Nonsense | LP |
| 112 | DMD | 8.9 | No | Yes |  | | negative | 51 | c.7402G>C | p.(Glu2468Gln) | Missense | VUS |
| 113 | DMD | 6.3 | No | Yes |  | | not done | 31 | c.4315A>T | p.(Arg1439*) | Nonsense | LP |
| 114 | DMD | 10.4 | No | Yes |  | | not done | 22 | c.2861G>A | p.(Trp954*) | Nonsense | LP |
| 115 | DMD | 10.5 | No | No | 7.8 | | not done | 40 | c.5636G>A | p.(Trp1879*) | Nonsense | LP |
| 116 | BMD | 4.0 | Yes | Yes |  | | not done | int40 | c.5740-16T>A |  | Splice-site | VUS |
| 117 | pending | 3 | Yes | Yes |  | | not done | 40 | c.5736_5739+1del |  | Splice-site | VUS |
| 118 | DMD | 12.3 | No | Yes |  | | not done | int51 | c.7542+1G>T |  | Splice-site | VUS |
| 119 | DMD | 5.1 | No | Yes |  | | not done | 9 | c.875del | p.(Pro292Leufs*43) | Frameshift | LP |
| 120 | DMD | 4.1 | No | Yes |  | | negative | 4 | c.236dup | p.(Ala80Glyfs*9) | Frameshift | LP |

P = Pathogenic; LP = Likely Pathogenic; VUS = Variants of Uncertain Significance; ACMG = American College of Medical Genetics and Genomics.

Patients No.17 and No.18 are first cousins.

Patients No.91 and No.92 are brothers.
