# Supplementary material for: Comprehensive analysis of 2097 patients with dystrophinopathy based on a database from 2011 to 2021
Source: Orphanet J Rare Dis. 2024 Aug 24;19:311. doi: 10.1186/s13023-024-03217-7 (PMC11344408; doi:10.1186/s13023-024-03217-7)
Supplement: Supplementary file 2 — Supplementary Material 2 [file 13023_2024_3217_MOESM2_ESM.docx]

Table S2. Clinical and dystrophin immunostaining data of patients with a previously reported small sequence variant.

| Patient | Phenotype | Age (y) | Family history | Ambulatory | Age (y)  at not ambulatory | Dystrophin immunostaining | Exon | Nucleotide change (NM_004006.3) | Predicted amino acid change | Predicted consequence | Predicted ACMG classification | | References |
| --- | --- | --- | --- | --- | --- | --- | --- | --- | --- | --- | --- | --- | --- |
| 1 | BMD | 11.6 | No | Yes |  | not done | 1 | c.31+5G>C |  | Splice-site | VUS | [1] | |
| 2 | BMD | 14.1 | No | Yes |  | not done | 1 | c.31+6T>C |  | Splice-site | VUS | [2] | |
| 3 | IMD | 11.6 | No | Yes |  | not done | 2 | c.70T>C | p.(Trp24Arg) | Missense | LP | [3] | |
| 4 | DMD | 10.4 | No | Yes |  | negative | int2 | c.93+1G>A |  | Splice-site | P | [4] | |
| 5 | DMD | 7.0 | No | Yes |  | negative | 3 | c.103C>T | p.(Gln35*) | Nonsense | P | [5] | |
| 6 | DMD | 9.6 | No | Yes |  | not done | int3 | c.186+1G>A |  | Splice-site | P | LOVD | |
| 7 | BMD | 10.9 | No | Yes |  | faint&patchy | int3 | c.186+2T>C |  | Splice-site | P | [6] | |
| 8 | pending | 4.3 | No | Yes |  | not done | int3 | c.186+1G>A |  | Splice-site | P | LOVD | |
| 9 | BMD | 6.0 | No | Yes |  | not done | int3 | c.187-2A>G |  | Splice-site | P | [7] | |
| 10 | BMD | 7.2 | No | Yes |  | faint&patchy | int3 | c.187-2A>G |  | Splice-site | P | [7] | |
| 11 | BMD | 6.7 | Yes | Yes |  | not done | 4 | c.196A>T | p.(Lys66*) | Nonsense | P | [8] | |
| 12 | DMD | 8.9 | No | Yes |  | not done | 4 | c.236dup | p.(Ala80Glyfs*9) | Frameshift | LP | LOVD | |
| 13 | BMD | 13.1 | Yes | Yes |  | faint&patchy | 4 | c.264_264+4delTGTAA |  | Splice-site | P | [9] | |
| 14 | DMD | 11.9 | No | No | 10.3 | not done | int4 | c.265-1G>A |  | Splice-site | LP | [10] | |
| 15 | DMD | 11.4 | No | Yes |  | negative | int5 | c.358-1G>T |  | Splice-site | P | [10] | |
| 16 | DMD | 6.5 | No | Yes |  | not done | int5 | c.357+1G>A |  | Splice-site | P | [11] | |
| 17 | BMD | 10.1 | No | Yes |  | faint&patchy | 6 | c.442A>C | p.(Thr148Pro) | Missense | VUS | [12] | |
| 18 | DMD | 12.6 | Unknown | Yes |  | not done | 6 | c.433C>T | p.(Arg145*) | Nonsense | P | [13] | |
| 19 | DMD | 9.1 | No | Yes |  | not done | 6 | c.433C>T | p.(Arg145*) | Nonsense | P | [13] | |
| 20 | DMD | 11.3 | Yes | Yes |  | not done | 6 | c.433C>T | p.(Arg145*) | Nonsense | P | [13] | |
| 21 | DMD | 13.3 | No | No | 9.2 | not done | 6 | c.433C>T | p.(Arg145*) | Nonsense | P | [13] | |
| 22 | IMD | 15.6 | No | Yes |  | faint&patchy | 6 | c.473A>T | p.(Asn158Ile) | Missense | LP | [14] | |
| 23 | DMD | 4.5 | No | Yes |  | not done | 7 | c.565C>T | p.(Gln189*) | Nonsense | P | [15] | |
| 24 | DMD | 16.8 | Yes | No | 12.7 | negative | 7 | c.583C>T | p.(Arg195*) | Nonsense | P | [16] | |
| 25 | DMD | 14.7 | No | No | 9.1 | not done | 7 | c.583C>T | p.(Arg195*) | Nonsense | P | [16] | |
| 26 | DMD | 6.0 | No | Yes |  | not done | 7 | c.583C>T | p.(Arg195*) | Nonsense | P | [16] | |
| 27 | DMD | 6.0 | Yes | Yes |  | not done | 7 | c.583C>T | p.(Arg195*) | Nonsense | P | [16] | |
| 28 | DMD | 10.5 | No | Yes |  | negative | int7 | c.649+5G>C |  | Splice-site | LP | [17] | |
| 29 | DMD | 4.9 | No | Yes |  | negative | int7 | c.649+2T>C |  | Splice-site | P | [18] | |
| 30 | DMD | 4.8 | No | Yes |  | not done | 8 | c.673A>T | p.(Lys225*) | Nonsense | P | [19] | |
| 31 | IMD | 10.4 | Yes | Yes |  | faint&patchy | 8 | c.711A>G | p.(Gln237Gln) | Synonymous | VUS | LOVD | |
| 32 | DMD | 15.2 | No | No | 11.6 | negative | 8 | c.724C>T | p.(Gln242*) | Nonsense | P | [20] | |
| 33 | DMD | 8.9 | No | Yes |  | not done | 8 | c.829C>T | p.(Gln277*) | Nonsense | P | [21] | |
| 34 | DMD | 5.1 | No | Yes |  | not done | 8 | c.829C>T | p.(Gln277*) | Nonsense | P | [21] | |
| 35 | DMD | 9.8 | No | Yes |  | negative | 8 | c.831+1G>C |  | Splice-site | P | LOVD | |
| 36 | pending | 2.1 | Yes | Yes |  | not done | 9 | c.850C>T | p.(Gln284*) | Nonsense | LP | [22] | |
| 37 | pending | 2.1 | Yes | Yes |  | not done | 9 | c.850C>T | p.(Gln284*) | Nonsense | LP | [22] | |
| 38 | IMD | 15.2 | No | Yes |  | faint&patchy | 9 | c.883C>T | p.(Arg295*) | Nonsense | LP | [23] | |
| 39 | DMD | 8.7 | No | Yes |  | negative | int9 | c.961-1G>A |  | Splice-site | P | [18] | |
| 40 | DMD | 15.6 | No | No | 11.8 | negative | 10 | c.1033C>T | p.(Gln345*) | Nonsense | P | [9] | |
| 41 | DMD | 10.0 | No | Yes |  | negative | 10 | c.1045G>T | p.(Glu349*) | Nonsense | P | LOVD | |
| 42 | pending | 2.0 | No | Yes |  | not done | 10 | c.1061G>A | p.(Trp354*) | Nonsense | LP | [24] | |
| 43 | DMD | 9.4 | No | Yes |  | not done | 10 | c.1099G>T | p.(Glu367*) | Nonsense | P | [25] | |
| 44 | DMD | 5.9 | Yes | Yes |  | not done | 10 | c.1132C>T | p.(Gln378*) | Nonsense | P | [6] | |
| 45 | DMD | 8.8 | Yes | Yes |  | negative | 10 | c.1149G>T | p.(Glu1829Asp) | Missense | VUS | [26] | |
| 46 | DMD | 5.6 | No | Yes |  | not done | 11 | c.1177C>T | p.(Gln393*) | Nonsense | P | [25] | |
| 47 | DMD | 4.7 | No | Yes |  | not done | 11 | c.1181del | p.(Gly394Alafs*13) | Frameshift | P | [27] | |
| 48 | DMD | 4.4 | No | Yes |  | not done | int11 | c.1332-9A>G |  | Splice-site | VUS | [28] | |
| 49 | DMD | 13.0 | Yes | No | 8 | not done | 12 | c.1412del | p.(Thr471Lysfs*16) | Frameshift | P | [9] | |
| 50 | DMD | 17.0 | No | No | 7.3 | negative | 13 | c.1594C>T | p.(Gln532*) | Nonsense | P | [15] | |
| 51 | DMD | 4.3 | No | Yes |  | not done | 13 | c.1510C>T | p.(Gln504*) | Nonsense | P | [10] | |
| 52 | pending | 4.9 | No | Yes |  | not done | 13 | c.1555G>T | p.(Glu519*) | Nonsense | LP | [29] | |
| 53 | DMD | 14.8 | No | No | 9.5 | negative | 14 | c.1615C>T | p.(Arg539*) | Nonsense | P | [30] | |
| 54 | DMD | 4.4 | No | Yes |  | not done | 14 | c.1615C>T | p.(Arg539*) | Nonsense | P | [30] | |
| 55 | DMD | 6.5 | No | Yes |  | not done | 14 | c.1620G>A | p.(Trp540*) | Nonsense | P | [31] | |
| 56 | DMD | 7.9 | Yes | Yes |  | not done | 14 | c.1642G>T | p.(Glu548*) | Nonsense | P | [23] | |
| 57 | DMD | 12.6 | No | No | 10.8 | not done | 14 | c.1683G>A | p.(Trp561*) | Nonsense | LP | [1] | |
| 58 | DMD | 6.9 | No | Yes |  | negative | 14 | c.1684C>T | p.(Gln562*) | Nonsense | P | [32] | |
| 59 | BMD | 11.4 | No | Yes |  | faint&patchy | int14 | c.1704+1G>C |  | Splice-site | P | [9] | |
| 60 | BMD | 9.6 | No | Yes |  | faint&patchy | int14 | c.1704+1G>T |  | Splice-site | P | [33] | |
| 61 | DMD | 13.8 | No | No | 10 | negative | 15 | c.1713insT | p.(Ser572*) | Nonsense | P | [9] | |
| 62 | DMD | 13.7 | No | Yes |  | negative | 15 | c.1777C>T | p.(Gln593*) | Nonsense | P | [34] | |
| 63 | DMD | 6.8 | No | Yes |  | negative | 15 | c.1793C>G | p.(Ser598*) | Nonsense | P | [35] | |
| 64 | BMD | 7.7 | No | Yes |  | not done | int15 | c.1812+1G>A |  | Splice-site | P | [6] | |
| 65 | BMD | 4.5 | No | Yes |  | not done | int15 | c.1812+1G>T |  | Splice-site | P | [36] | |
| 66 | DMD | 14.3 | No | No | 9.8 | negative | 16 | c.1886C>G | p.(Ser629*) | Nonsense | P | [9] | |
| 67 | DMD | 6.5 | No | Yes |  | not done | 16 | c.1901dup | p.(Ser635Valfs*12) | Frameshift | P | [37] | |
| 68 | DMD | 6.5 | Yes | Yes |  | not done | 16 | c.1928G>A | p.(Trp643*) | Nonsense | P | [38] | |
| 69 | DMD | 8.9 | Unknown | Yes |  | not done | 17 | c.2125C>T | p.(Gln709*) | Nonsense | P | [27] | |
| 70 | DMD | 14.0 | No | No | 9.5 | negative | 17 | c.2137C>T | p.(Gln713*) | Nonsense | P | [9] | |
| 71 | BMD | 19.9 | No | Yes |  | faint&patchy | int17 | c.2169-1G>T |  | Splice-site | P | [9] | |
| 72 | DMD | 15.1 | No | No | 9 | negative | 18 | c.2191del | p.(Leu731Phefs*29) | Frameshift | P | [9] | |
| 73 | DMD | 13.5 | No | Yes |  | not done | 18 | c.2213C>G | p.(Ser738*) | Nonsense | P | [26] | |
| 74 | DMD | 9.1 | No | Yes |  | not done | 18 | c.2227C>T | p.(Gln743*) | Nonsense | P | [10] | |
| 75 | DMD | 19.0 | No | No | 13.9 | negative | 19 | c.2308A>T | p.(Lys770*) | Nonsense | P | [13] | |
| 76 | DMD | 17.3 | No | No | 11 | negative | 19 | c.2302C>T | p.(Arg768*) | Nonsense | P | [39] | |
| 77 | DMD | 13.0 | No | Yes |  | negative | 19 | c.2302C>T | p.(Arg768*) | Nonsense | P | [39] | |
| 78 | DMD | 15.6 | No | No | 11.6 | negative | 19 | c.2300del | p.(Glu767Glyfs*26) | Frameshift | P | [9] | |
| 79 | DMD | 11.2 | Yes | Yes |  | negative | 19 | c.2380G>A | p.(Glu794Lys) | Missense | VUS | [40] | |
| 80 | DMD | 9.9 | No | Yes |  | negative | 19 | c.2380G>T | p.(Gln794*) | Nonsense | P | [27] | |
| 81 | DMD | 6.0 | No | Yes |  | not done | int19 | c.2380+1G>T |  | Splice-site | P | [26] | |
| 82 | DMD | 8.7 | Unknown | Yes |  | not done | 20 | c.2407C>T | p.(Gln803*) | Nonsense | P | [19] | |
| 83 | DMD | 7.9 | No | Yes |  | negative | 20 | c.2436G>A | p.(Trp812*) | Nonsense | P | [36] | |
| 84 | DMD | 7.4 | No | Yes |  | not done | 20 | c.2525T>A | p.(Leu842*) | Nonsense | LP | LOVD | |
| 85 | DMD | 16.3 | No | No | 11.5 | not done | 20 | c.2530C>T | p.(Gln844*) | Nonsense | P | [12] | |
| 86 | DMD | 4.4 | No | Yes |  | not done | int20 | c.2622+1G>T |  | Splice-site | P | [41] | |
| 87 | IMD | 12.3 | Yes | Yes |  | faint&patchy | int20 | c.2623-2A>C |  | Splice-site | P | [42] | |
| 88 | IMD | 11.9 | Yes | Yes |  | not done | int20 | c.2623-2A>C |  | Splice-site | P | [42] | |
| 89 | DMD | 8.6 | No | Yes |  | negative | 21 | c.2656C>T | p.(Gln886*) | Nonsense | P | [43] | |
| 90 | DMD | 14.5 | No | No | 8.1 | negative | 21 | c.2665C>T | p.(Arg889*) | Nonsense | P | [6] | |
| 91 | DMD | 15.6 | No | No | 11 | negative | 21 | c.2665C>T | p.(Arg889*) | Nonsense | P | [6] | |
| 92 | DMD | 7.6 | No | Yes |  | not done | 21 | c.2665C>T | p.(Arg889*) | Nonsense | LP | [6] | |
| 93 | DMD | 9.1 | No | Yes |  | not done | 21 | c.2758C>T | p.(Gln920*) | Nonsense | P | [35] | |
| 94 | DMD | 8.4 | Unknown | No | 8.1 | not done | 21 | c.2776C>T | p.(Gln926*) | Nonsense | P | [29] | |
| 95 | DMD | 4.1 | No | Yes |  | not done | int21 | c.2803+1G>A |  | Splice-site | P | [44] | |
| 96 | DMD | 17.8 | Yes | No | 9.7 | negative | int21 | c.2803+1G>A |  | Splice-site | P | [44] | |
| 97 | pending | 4.1 | No | Yes |  | not done | int21 | c.2803+1G>A |  | Splice-site | LP | [44] | |
| 98 | DMD | 16.4 | Yes | No | 9.3 | negative | 22 | c.2816T>A | p.(Leu939*) | Nonsense | P | [9] | |
| 99 | DMD | 13.6 | Yes | No | 10 | not done | 22 | c.2832T>A | p.(Tyr944*) | Nonsense | P | [34] | |
| 100 | DMD | 19.6 | No | No | 12 | negative | int22 | c.2949+1G>A |  | Splice-site | P | [45] | |
| 101 | DMD | 5.0 | No | Yes |  | negative | int22 | c.2949+1G>T |  | Splice-site | P | [46] | |
| 102 | DMD | 4.8 | No | Yes |  | not done | 23 | c.3151C>T | p.(Arg1051*) | Nonsense | P | [47] | |
| 103 | DMD | 3.1 | No | Yes |  | not done | 23 | c.3151C>T | p.(Arg1051*) | Nonsense | P | [47] | |
| 104 | DMD | 6.2 | No | Yes |  | not done | 23 | c.3151C>T | p.(Arg1051*) | Nonsense | P | [47] | |
| 105 | DMD | 8.9 | No | No | 7.9 | not done | 23 | c.3151C>T | p.(Arg1051*) | Nonsense | P | [47] | |
| 106 | DMD | 5.6 | No | Yes |  | not done | 23 | c.3151C>T | p.(Arg1051*) | Nonsense | P | [47] | |
| 107 | DMD | 7.9 | No | Yes |  | not done | 23 | c.3151C>T | p.(Arg1051*) | Nonsense | P | [47] | |
| 108 | DMD | 5.0 | Yes | Yes |  | not done | 24 | c.3168del | p.(His1056Glnfs*5) | Frameshift | P | [48] | |
| 109 | DMD | 17.1 | No | No | 10.3 | negative | 24 | c.3257dup | p.(Gln1087Alafs*11) | Frameshift | P | [49] | |
| 110 | DMD | 6.2 | No | Yes |  | not done | 24 | c.3259C>T | p.(Gln1087*) | Nonsense | P | [24] | |
| 111 | DMD | 11.3 | No | No | 10.4 | not done | int24 | c.3277-1G>A |  | Splice-site | P | [50] | |
| 112 | pending | 2.3 | Yes | Yes |  | not done | 25 | c.3328G>T | p.(Glu1110*) | Nonsense | LP | [51] | |
| 113 | BMD | 9.1 | Yes | Yes |  | faint&patchy | 25 | c.3337C>T | p.(Gln1113*) | Nonsense | P | [35] | |
| 114 | BMD | 12.9 | No | Yes |  | faint&patchy | 25 | c.3388G>T | p.(Glu1130*) | Nonsense | P | [9] | |
| 115 | BMD | 13.4 | No | Yes |  | faint&patchy | 25 | c.3432G>T | p.(Gln1144His) | Missense | VUS | [52] | |
| 116 | BMD | 8.8 | No | Yes |  | not done | 25 | c.3432+1G>A |  | Splice-site | P | [53] | |
| 117 | DMD | 5.2 | No | Yes |  | not done | 26 | c.3516G>A | p.(Trp1172*) | Nonsense | P | [7] | |
| 118 | DMD | 5.4 | No | Yes |  | not done | 26 | c.3523C>T | p.(Gln1175*) | Nonsense | P | LOVD | |
| 119 | DMD | 11.9 | No | No | 9.6 | not done | 26 | c.3544G>T | p.(Glu1182*) | Nonsense | P | [35] | |
| 120 | DMD | 7.4 | No | Yes |  | negative | 26 | c.3562A>T | p.(Lys1188*) | Nonsense | P | [54] | |
| 121 | DMD | 7.1 | No | Yes |  | negative | 26 | c.3580C>T | p.(Gln1194*) | Nonsense | P | [54] | |
| 122 | pending | 1.5 | No | Yes |  | not done | 26 | c.3603+1G>T |  | Splice-site | LP | [29] | |
| 123 | DMD | 10.3 | No | No |  | negative | int26 | c.3603+1G>T |  | Splice-site | P | [29] | |
| 124 | DMD | 6.3 | Yes | Yes |  | not done | int26 | c.3603+1G>T |  | Splice-site | P | [29] | |
| 125 | DMD | 5.3 | No | Yes |  | not done | int26 | c.3603+2T>A |  | Splice-site | P | [55] | |
| 126 | pending | 3.2 | Yes | Yes |  | not done | int26 | c.3603+2dup |  | Splice-site | VUS | [1] | |
| 127 | DMD | 12.2 | No | Yes |  | not done | 27 | c.3622C>T | p.(Gln1208*) | Nonsense | P | [35] | |
| 128 | DMD | 5.3 | No | Yes |  | not done | 27 | c.3625C>T | p.(Gln1209*) | Nonsense | P | [27] | |
| 129 | DMD | 8.4 | No | Yes |  | not done | 27 | c.3715G>T | p.(Glu1239*) | Nonsense | P | [49] | |
| 130 | DMD | 14.0 | No | No | 8 | negative | 27 | c.3747G>A | p.(Trp1249*) | Nonsense | P | [56] | |
| 131 | DMD | 13.8 | Yes | Yes |  | negative | int27 | c.3786+1G>T |  | Splice-site | P | [50] | |
| 132 | DMD | 9.8 | Yes | Yes |  | not done | int27 | c.3786+1G>T |  | Splice-site | P | [50] | |
| 133 | DMD | 7.6 | No | Yes |  | not done | int27 | c.3786+1G>T |  | Splice-site | P | [50] | |
| 134 | DMD | 9.3 | No | Yes |  | negative | int27 | c.3786+5G>C |  | Splice-site | VUS | [57] | |
| 135 | DMD | 4.5 | No | Yes |  | not done | 28 | c.3795G>A | p.(Trp1265*) | Nonsense | P | [32] | |
| 136 | BMD | 12.5 | No | Yes |  | faint&patchy | 29 | c.3940C>T | p.(Arg1314*) | Nonsense | P | [27] | |
| 137 | DMD | 10.8 | No | No | 10.2 | negative | 30 | c.4108C>T | p.(Gln1370*) | Nonsense | P | [30] | |
| 138 | DMD | 8.5 | No | Yes |  | negative | 30 | c.4117C>T | p.(Gln1373*) | Nonsense | P | [27] | |
| 139 | DMD | 6.0 | No | Yes |  | negative | 30 | c.4120G>T | p.(Glu1374*) | Nonsense | P | [58] | |
| 140 | DMD | 17.3 | No | No | 11.6 | not done | 30 | c.4150G>T | p.(Glu1384*) | Nonsense | LP | [59] | |
| 141 | DMD | 9.9 | No | No | 8.7 | negative | 30 | c.4231C>T | p.(Gln1411*) | Nonsense | P | [60] | |
| 142 | DMD | 7.0 | Unknown | Yes |  | not done | 31 | c.4290_4291del | p.(His1430Glnfs*15) | Frameshift | P | [23] | |
| 143 | DMD | 10.4 | No | No | 9.1 | negative | 32 | c.4375C>T | p.(Arg1459*) | Nonsense | P | [61] | |
| 144 | DMD | 10.5 | No | Yes |  | negative | 32 | c.4414C>T | p.(Gln1472*) | Nonsense | P | [13] | |
| 145 | DMD | 14.3 | Yes | No | 12.6 | not done | 32 | c.4414C>T | p.(Gln1472*) | Nonsense | P | [13] | |
| 146 | DMD | 11.2 | No | No | 10.5 | negative | 32 | c.4483C>T | p.(Gln1495*) | Nonsense | P | [35] | |
| 147 | DMD | 8.7 | No | Yes |  | not done | 32 | c.4483C>T | p.(Gln1495*) | Nonsense | P | [35] | |
| 148 | DMD | 7.6 | No | Yes |  | negative | 32 | c.4518G>A | p.(Val1506Val) | Synonymous | LP | [35] | |
| 149 | DMD | 11.6 | No | Yes |  | negative | 33 | c.4538_4541del | p.(Ser1513Lysfs*2) | Frameshift | P | [62] | |
| 150 | DMD | 12.5 | No | No | 10.9 | negative | 33 | c.4576G>T | p.(Gly1526*) | Nonsense | P | LOVD | |
| 151 | DMD | 5.0 | No | Yes |  | not done | 33 | c.4618G>T | p.(Glu1540*) | Nonsense | P | [35] | |
| 152 | DMD | 16.0 | No | No | 10.4 | negative | 33 | c.4630del | p.(Arg1544Glufs*2) | Frameshift | P | [9] | |
| 153 | DMD | 7.3 | No | Yes |  | negative | 33 | c.4660G>T | p.(Glu1554*) | Nonsense | P | [34] | |
| 154 | DMD | 8.3 | No | Yes |  | not done | 34 | c.4675-2A>G |  | Splice-site | P | [44] | |
| 155 | DMD | 4.1 | No | Yes |  | negative | 34 | c.4690C>T | p.(Gln1564*) | Nonsense | P | [27] | |
| 156 | DMD | 15.7 | No | No | 10.1 | negative | 34 | c.4729C>T | p.(Arg1577*) | Nonsense | P | [54] | |
| 157 | DMD | 14.1 | No | No | 12.3 | not done | 34 | c.4729C>T | p.(Arg1577*) | Nonsense | P | [54] | |
| 158 | DMD | 13.0 | No | No | 10.3 | negative | 34 | c.4746_4747del | p.(Leu1583Aspfs*18) | Frameshift | P | [63] | |
| 159 | DMD | 23.3 | Yes | No | 9 | negative | 34 | c.4808_4809insGGAA | p.(Pro1604Glufs*3) | Frameshift | P | [9] | |
| 160 | DMD | 9.5 | No | Yes |  | not done | 34 | c.4841del | p. (Gly1614Glufs*15) | Frameshift | P | [30] | |
| 161 | BMD | 8.0 | No | Yes |  | faint&patchy | int34 | c.4845+2T>G |  | Splice-site | P | ClinVar | |
| 162 | DMD | 9.7 | No | Yes |  | not done | 35 | c.4856_4857del | p.(Lys1619Argfs*3) | Frameshift | P | [64] | |
| 163 | DMD | 12.2 | No | No | 9.3 | not done | 35 | c.4996C>T | p.(Arg1666*) | Nonsense | P | [15] | |
| 164 | DMD | 10.3 | No | Yes |  | not done | 36 | c.5089C>T | p.(Gln1697*) | Nonsense | P | [65] | |
| 165 | DMD | 12.0 | No | No | 9 | negative | 36 | c.5118_5119del | p.(Lys1708Glufs*10) | Frameshift | P | [18] | |
| 166 | DMD | 4.4 | No | Yes |  | not done | 37 | c.5161A>T | p.(Lys1721*) | Nonsense | P | [17] | |
| 167 | DMD | 7.7 | No | Yes |  | negative | 37 | c.5266C>T | p.(Gln1756*) | Nonsense | P | [59] | |
| 168 | DMD | 11.8 | No | Yes |  | not done | 38 | c.5444A>G | p.(Asp1815Gly) | Missense | LP | [10] | |
| 169 | DMD | 4.8 | No | Yes |  | not done | 39 | c.5452G>T | p.(Glu1818*) | Nonsense | P | [66] | |
| 170 | DMD | 6.1 | No | Yes |  | negative | 39 | c.5452G>T | p.(Glu1818*) | Nonsense | P | [66] | |
| 171 | DMD | 11.0 | No | No | 9.9 | negative | 39 | c.5485C>T | p.(Gln1829*) | Nonsense | P | [9] | |
| 172 | DMD | 5.1 | Yes | Yes |  | not done | 39 | c.5530C>T | p.(Arg1844*) | Nonsense | P | [44] | |
| 173 | DMD | 13.6 | No | No | 10 | negative | 39 | c.5530C>T | p.(Arg1844*) | Nonsense | P | [44] | |
| 174 | DMD | 8.8 | No | Yes |  | negative | 39 | c.5563C>T | p.(Gln1855*) | Nonsense | P | [45] | |
| 175 | DMD | 6.4 | Yes | Yes |  | not done | 40 | c.5602_5605del | p.(Arg1868Glufs*5) | Frameshift | P | [1] | |
| 176 | DMD | 6.6 | Yes | Yes |  | not done | 40 | c.5602_5605del | p.(Arg1868Glufs*5) | Frameshift | P | [1] | |
| 177 | IMD | 13.2 | No | Yes |  | not done | 40 | c.5653C>T | p.(Gln885*) | Nonsense | P | [35] | |
| 178 | IMD | 11.3 | No | Yes |  | not done | 40 | c.5653C>T | p.(Gln885*) | Nonsense | P | [35] | |
| 179 | DMD | 11.1 | Yes | Yes |  | negative | 40 | c.5697dup | p.(Leu1900Ilefs*6) | Frameshift | P | [67] | |
| 180 | DMD | 11.3 | No | No | 10.2 | not done | 41 | c.5868G>A | p.(Trp1956*) | Nonsense | P | [25] | |
| 181 | DMD | 20.8 | Yes | No | 8 | not done | 41 | c.5899C>T | p.(Arg1967*) | Nonsense | P | [37] | |
| 182 | DMD | 10.6 | No | Yes |  | not done | 41 | c.5899C>T | p.(Arg1967*) | Nonsense | P | [37] | |
| 183 | DMD | 5.1 | No | Yes |  | not done | 41 | c.5899C>T | p.(Arg1967*) | Nonsense | P | [37] | |
| 184 | DMD | 6.3 | No | Yes |  | not done | 41 | c.5899C>T | p.(Arg1967*) | Nonsense | P | [37] | |
| 185 | DMD | 5.5 | Yes | Yes |  | not done | 41 | c.5899C>T | p.(Arg1967*) | Nonsense | P | [37] | |
| 186 | BMD | 14.7 | Yes | Yes |  | not done | int41 | c.5922+5G>A |  | Splice-site | VUS | LOVD | |
| 187 | DMD | 7.1 | No | Yes |  | not done | 42 | c.6006_6007del | p.(Glu2003Asnfs*19) | Frameshift | P | [34] | |
| 188 | DMD | 18.5 | No | No | 11.1 | negative | 42 | c.6033insTTAA | p.(Glu2013Ilefs*11) | Frameshift | P | [9] | |
| 189 | DMD | 9.6 | No | Yes |  | negative | 42 | c.6045del | p.(Glu2015Aspfs*24) | Frameshift | P | [9] | |
| 190 | DMD | 10.1 | No | Yes |  | not done | 42 | c.6072T>A | p.(Cys2024*) | Nonsense | P | [45] | |
| 191 | DMD | 9.9 | No | Yes |  | negative | 43 | c.6127del | p.(Asp2043Ilefs*30) | Frameshift | P | [10] | |
| 192 | DMD | 16.9 | No | No | 10.6 | not done | 43 | c.6223C>T | p.(Gln2075*) | Nonsense | P | [6] | |
| 193 | DMD | 10.4 | Yes | Yes |  | negative | 43 | c.6283C>T | p.(Arg2095*) | Nonsense | P | [68] | |
| 194 | DMD | 4.3 | No | Yes |  | not done | 43 | c.6283C>T | p.(Arg2095*) | Nonsense | P | [68] | |
| 195 | DMD | 8.8 | No | Yes |  | not done | 43 | c.6290+1G>A |  | Splice-site | P | [23] | |
| 196 | DMD | 24.5 | No | No | 12 | negative | 44 | c.6292C>T | p.(Arg2098*) | Nonsense | P | [13] | |
| 197 | DMD | 13.9 | No | No | 10 | not done | 44 | c.6391_6392del | p.(Gln2131Asnfs*3) | Frameshift | P | [69] | |
| 198 | DMD | 14.9 | No | Yes |  | negative | 44 | c.6429G>A | p.(Trp2143*) | Nonsense | P | [6] | |
| 199 | DMD | 4.0 | No | Yes |  | not done | 44 | c.6408G>A | p.(Trp2136*) | Nonsense | P | [26] | |
| 200 | DMD | 10.0 | No | No | 7.8 | not done | 44 | c.6430dup | p.(Tyr2144Leufs*3) | Frameshift | P | [32] | |
| 201 | DMD | 7.0 | No | Yes |  | not done | int44 | c.6438+1G>T |  | Splice-site | P | [35] | |
| 202 | DMD | 7.8 | No | Yes |  | negative | 45 | c.6577C>T | p.(Gln2193*) | Nonsense | P | [14] | |
| 203 | DMD | 9.8 | No | No | 9.3 | negative | 45 | c.6577C>T | p.(Gln2193*) | Nonsense | P | [14] | |
| 204 | DMD | 4.7 | Yes | Yes |  | not done | 45 | c.6592C>T | p.(Gln2198*) | Nonsense | P | [70] | |
| 205 | DMD | 5.5 | No | Yes |  | negative | 45 | c.6567del | p.(Leu2190Cysfs*17) | Frameshift | P | [48] | |
| 206 | DMD | 12.3 | No | Yes |  | not done | 45 | c.6611dup | p.(Arg2205Glufs*18) | Frameshift | P | [7] | |
| 207 | DMD | 9.1 | No | Yes |  | not done | int45 | c.6615-2A>G |  | Splice-site | P | [71] | |
| 208 | DMD | 13.7 | No | No | 12 | not done | 46 | c.6651_6652del | p.(Asp2219Phefs*3) | Frameshift | P | [72] | |
| 209 | IMD | 5.6 | No | Yes |  | faint&patchy | 46 | c.6762G>A | p.(Lys2254Lys) | synonymous | VUS | LOVD | |
| 210 | DMD | 15.9 | No | No | 9 | negative | int46 | c.6762+1G>A |  | Splice-site | P | [23] | |
| 211 | DMD | 7.5 | No | Yes |  | not done | int46 | c.6762+1G>A |  | Splice-site | P | [23] | |
| 212 | DMD | 9.6 | Yes | Yes |  | not done | 47 | c.6804_6807del | p.(Lys2268Asnfs*2) | Frameshift | P | [35] | |
| 213 | DMD | 10.7 | No | No | 10.5 | negative | 47 | c.6826_6866del | p.(Pro2276*) | Nonsense | P | [73] | |
| 214 | DMD | 16.1 | Yes | No | 10.3 | negative | 48 | c.7029G>A | p.(Trp2343*) | Nonsense | P | [74] | |
| 215 | DMD | 5.7 | No | Yes |  | not done | 48 | c.7066C>T | p.(Gln2356*) | Nonsense | P | [75] | |
| 216 | DMD | 5.1 | Yes | Yes |  | not done | 48 | c.7098+1G>A |  | Splice-site | P | [13] | |
| 217 | BMD | 21.7 | No | Yes |  | faint&patchy | 49 | c.7159C>T | p.(Gln2387*) | Nonsense | P | [76] | |
| 218 | DMD | 8.9 | No | Yes |  | negative | int51 | c.7310-19A>G |  | Splice-site | LP | [77] | |
| 219 | DMD | 13.5 | No | No | 10.2 | negative | 51 | c.7327_7328insA | p.(Thr2443Asnfs*10) | Frameshift | P | [9] | |
| 220 | DMD | 13.9 | No | No | 11.4 | negative | 52 | c.7657C>T | p.(Arg2553*) | Nonsense | P | [78] | |
| 221 | DMD | 10.9 | No | No | 7.6 | negative | 52 | c.7657C>T | p.(Arg2553*) | Nonsense | P | [78] | |
| 222 | DMD | 8.2 | No | Yes |  | not done | 52 | c.7657C>T | p.(Arg2553*) | Nonsense | P | [78] | |
| 223 | DMD | 6.7 | No | Yes |  | not done | int52 | c.7661-2A>G |  | Splice-site | P | [49] | |
| 224 | DMD | 8.0 | No | Yes |  | negative | 53 | c.7755G>A | p.(Trp2585*) | Nonsense | P | [53] | |
| 225 | DMD | 6.8 | Yes | Yes |  | not done | 53 | c.7817G>A | p.(Trp2606*) | Nonsense | P | [32] | |
| 226 | DMD | 13.1 | No | No | 10.1 | not done | 54 | c.7899G>A | p.(Trp2633*) | Nonsense | P | [79] | |
| 227 | DMD | 13.4 | No | No | 10.4 | negative | int54 | c.8028-1G>C |  | Splice-site | P | [9] | |
| 228 | DMD | 12.9 | Yes | Yes |  | negative | int54 | c.8027+2T>C |  | Splice-site | P | [80] | |
| 229 | DMD | 7.9 | No | Yes |  | not done | int54 | c.8027+1G>A |  | Splice-site | P | [6] | |
| 230 | DMD | 13.7 | No | No | 7.5 | negative | 55 | c.8038C>T | p.(Arg2680*) | Nonsense | P | [30] | |
| 231 | DMD | 4.5 | No | Yes |  | not done | 55 | c.8038C>T | p.(Arg2680*) | Nonsense | P | [30] | |
| 232 | DMD | 9.9 | No | Yes |  | negative | 55 | c.8087delT | p.(Leu2696Argfs*30) | Frameshift | P | [81] | |
| 233 | DMD | 11.9 | No | No | 8.2 | not done | int55 | c.8217+1G>A |  | Splice-site | LP | [82] | |
| 234 | DMD | 8.9 | No | Yes |  | not done | int55 | c.8218-2A>G |  | Splice-site | P | [35] | |
| 235 | DMD | 6.8 | No | Yes |  | not done | int55 | c.8218-2A>G |  | Splice-site | P | [35] | |
| 236 | DMD | 11.3 | No | No | 8 | not done | 56 | c.8299G>T | p.(Gln2767*) | Nonsense | LP | [8] | |
| 237 | DMD | 5.7 | No | Yes |  | not done | int56 | c.8390+1G>A |  | Splice-site | P | [49] | |
| 238 | DMD | 12.3 | Unknown | No | 11.1 | negative | 57 | c.8420G>A | p.(Trp2807*) | Nonsense | P | [1] | |
| 239 | DMD | 8.5 | No | Yes |  | not done | 58 | c.8608C>T | p.(Arg2870*) | Nonsense | P | [15] | |
| 240 | DMD | 6.8 | No | Yes |  | not done | 58 | c.8608C>T | p.(Arg2870*) | Nonsense | P | [15] | |
| 241 | DMD | 10.2 | No | Yes |  | not done | 58 | c.8608C>T | p.(Arg2870*) | Nonsense | P | [15] | |
| 242 | DMD | 7.3 | No | Yes |  | negative | 58 | c.8608C>T | p.(Arg2870*) | Nonsense | P | [15] | |
| 243 | DMD | 16.5 | Yes | No | 14.4 | negative | 59 | c.8713C>T | p.(Arg2905*) | Nonsense | P | [61] | |
| 244 | DMD | 10.2 | No | Yes |  | not done | 59 | c.8713C>T | p.(Arg2905*) | Nonsense | P | [61] | |
| 245 | DMD | 19.5 | Yes | No | 7 | not done | 59 | c.8713C>T | p.(Arg2905*) | Nonsense | P | [61] | |
| 246 | DMD | 15.9 | Yes | No | 8 | negative | 59 | c.8740G>T | p.(Glu2914*) | Nonsense | P | [9] | |
| 247 | DMD | 9.3 | Yes | Yes |  | negative | 59 | c.8775G>A | p.(Trp2925*) | Nonsense | P | [35] | |
| 248 | DMD | 6.4 | Yes | Yes |  | not done | 59 | c.8775G>A | p.(Trp2925*) | Nonsense | P | [35] | |
| 249 | DMD | 13.9 | No | No | 9 | not done | 59 | c.8818C>T | p.(Gln2940*) | Nonsense | LP | [50] | |
| 250 | DMD | 11.5 | No | Yes |  | negative | 59 | c.8910_8913del | p.(Leu2971Lysfs*17) | Frameshift | P | [83] | |
| 251 | DMD | 20.5 | Yes | No | 12 | negative | 60 | c.8944C>T | p.(Arg2982*) | Nonsense | P | [84] | |
| 252 | DMD | 9.8 | No | Yes |  | negative | 60 | c.8944C>T | p.(Arg2982*) | Nonsense | P | [84] | |
| 253 | DMD | 9.6 | Yes | No | 8.1 | not done | 60 | c.8944C>T | p.(Arg2982*) | Nonsense | P | [84] | |
| 254 | DMD | 11.7 | No | No | 9.1 | not done | 61 | c.9100C>T | p.(Arg3034*) | Nonsense | P | [85] | |
| 255 | DMD | 8.2 | No | Yes |  | not done | 61 | c.9100C>T | p.(Arg3034*) | Nonsense | P | [85] | |
| 256 | DMD | 9.7 | No | Yes |  | negative | int62 | c.9224+1G>C |  | Splice-site | P | [1] | |
| 257 | IMD | 9.3 | No | Yes |  | not done | int62 | c.9225-647A>G |  | Splice-site | VUS | [86] | |
| 258 | DMD | 9.4 | No | No | 7.4 | not done | 63 | c.9248G>A | p.(Trp3083*) | Nonsense | P | [87] | |
| 259 | DMD | 12.2 | No | No | 10.9 | negative | 64 | c.9297_9300dup | p.(Val3101*) | Nonsense | P | [9] | |
| 260 | DMD | 11.4 | Yes | Yes |  | negative | 64 | c.9297_9300dup | p.(Val3101*) | Nonsense | P | [9] | |
| 261 | DMD | 10.7 | No | No | 10.3 | negative | 64 | c.9337C>T | p.(Arg3113*) | Nonsense | P | [86] | |
| 262 | DMD | 17.5 | No | No | 10.8 | negative | 64 | c.9337C>T | p.(Arg3113*) | Nonsense | P | [86] | |
| 263 | DMD | 7.6 | No | Yes |  | not done | 64 | c.9337C>T | p.(Arg3113*) | Nonsense | P | [86] | |
| 264 | DMD | 4.2 | No | Yes |  | not done | 64 | c.9337C>T | p.(Arg3113*) | Nonsense | P | [86] | |
| 265 | DMD | 9.2 | No | Yes |  | not done | 64 | c.9358_9359insA | p.(Cys3120*) | Nonsense | P | LOVD | |
| 266 | DMD | 8.0 | Unknown | Yes |  | negative | int64 | c.9361+1G>C |  | Splice-site | P | [88] | |
| 267 | DMD | 4.6 | No | Yes |  | not done | 65 | c.9454_9457del | p.(Asn3152Valfs*2) | Frameshift | P | [26] | |
| 268 | DMD | 4.8 | No | Yes |  | not done | 65 | c.9563+1G>A |  | Splice-site | P | [89] | |
| 269 | DMD | 17.3 | No | No | 12.7 | negative | 66 | c.9568C>T | p.(Arg3190*) | Nonsense | P | [90] | |
| 270 | DMD | 7.5 | Yes | Yes |  | not done | 66 | c.9568C>T | p.(Arg3190*) | Nonsense | P | [90] | |
| 271 | DMD | 5.5 | Yes | Yes |  | not done | 66 | c.9568C>T | p.(Arg3190*) | Nonsense | P | [90] | |
| 272 | DMD | 11.4 | Yes | No | 9 | negative | 67 | c.9722_9723del | p.(Ser3241Tyrfs*9) | Frameshift | P | [9] | |
| 273 | DMD | 12.7 | No | No | 10 | negative | int67 | c.9807+5G>A |  | Splice-site | LP | [47] | |
| 274 | DMD | 11.6 | Yes | Yes |  | negative | int67 | c.9807+5G>A |  | Splice-site | LP | [47] | |
| 275 | DMD | 12.5 | No | No | 10.8 | negative | int68 | c.9974+1G>A |  | Splice-site | P | [35] | |
| 276 | DMD | 9.7 | Yes | Yes |  | not done | 69 | c.10027del | p.(Ser3343Leufs*34) | Frameshift | P | [14] | |
| 277 | DMD | 9.5 | No | Yes |  | negative | int69 | c.10087-1G>C |  | Splice-site | P | [43] | |
| 278 | DMD | 5.4 | No | Yes |  | negative | int69 | c.10086+1G>A |  | Splice-site | P | [91] | |
| 279 | DMD | 5.9 | Yes | Yes |  | not done | 70 | c.10108C>T | p.(Arg3370*) | Nonsense | P | [30] | |
| 280 | DMD | 13.0 | No | Yes |  | negative | 70 | c.10108C>T | p.(Arg3370*) | Nonsense | P | [30] | |
| 281 | DMD | 9.8 | No | Yes |  | negative | 70 | c.10108C>T | p.(Arg3370*) | Nonsense | P | [30] | |
| 282 | DMD | 8.8 | No | Yes |  | negative | 70 | c.10108C>T | p.(Arg3370*) | Nonsense | P | [30] | |
| 283 | DMD | 4.8 | No | Yes |  | not done | 70 | c.10108C>T | p.(Arg3370*) | Nonsense | P | [30] | |
| 284 | DMD | 4.9 | No | Yes |  | not done | 70 | c.10141C>T | p.(Arg3381*) | Nonsense | P | [89] | |
| 285 | DMD | 4.8 | No | Yes |  | not done | 70 | c.10141C>T | p.(Arg3381*) | Nonsense | P | [89] | |
| 286 | DMD | 4.7 | No | Yes |  | not done | 70 | c.10171C>T | p.(Arg3391*) | Nonsense | P | [92] | |
| 287 | DMD | 17.3 | Yes | No | 10.5 | negative | 70 | c.10171C>T | p.(Arg3391*) | Nonsense | P | [92] | |
| 288 | DMD | 4.0 | No | Yes |  | not done | 70 | c.10171C>T | p.(Arg3391*) | Nonsense | P | [92] | |
| 289 | DMD | 6.2 | No | Yes |  | not done | 70 | c.10171C>T | p.(Arg3391*) | Nonsense | P | [92] | |
| 290 | DMD | 4.0 | No | Yes |  | not done | int70 | c.10223+1G>A |  | Splice-site | P | [8] | |
| 291 | pending | 4.4 | No | Yes |  | not done | 72 | c.10279C>T | p.(Gln3427*) | Nonsense | P | [26] | |
| 292 | BMD | 13.4 | No | Yes |  | faint&patchy | 74 | c.10429C>T | p.(Gln3477*) | Nonsense | P | LOVD | |
| 293 | DMD | 15.8 | No | Yes |  | negative | 74 | c.10453_10454del | p.(Leu3485Glufs*5) | Frameshift | P | [93] | |
| 294 | BMD | 8.8 | No | Yes |  | not done | 74 | c.10453del | p.(Leu3485*) | Nonsense | P | [6] | |
| 295 | DMD | 13.0 | No | Yes |  | negative | 74 | c.10454del | p.(Leu3485Argfs*11) | Frameshift | P | [84] | |
| 296 | DMD | 11.5 | Yes | Yes |  | negative | 74 | c.10454del | p.(Leu3485Argfs*11) | Frameshift | P | [84] | |
| 297 | BMD | 13.2 | No | Yes |  | not done | 74 | c.10454del | p.(Leu3485Argfs*11) | Frameshift | P | [84] | |
| 298 | BMD | 12.0 | No | Yes |  | faint&patchy | 74 | c.10543G>T | p.(Glu3515*) | Nonsense | P | [44] | |

P = Pathogenic; LP = Likely Pathogenic; VUS = Variants of Uncertain Significance; ACMG = American College of Medical Genetics and Genomics.

LOVD indicates variants that have been previously reported in the Leiden Open Variantion Database (https://databases.lovd.nl).

ClinVar indicates variants that have been previously reported in the ClinVar Database (https://www.ncbi.nlm.nih.gov/clinvar).

Patients No. 36 and No. 37, No. 87 and No. 88, No. 243 and No. 245, and No. 247 and No. 248 are first cousins.

Patients No.177 and No.178, No.251 and No.253 are brothers.

Patient No.259 is No.260’s uncle.

**References**

1. Deburgrave, N., et al., Protein- and mRNA-based phenotype-genotype correlations in DMD/BMD with point mutations and molecular basis for BMD with nonsense and frameshift mutations in the DMD gene. Hum Mutat, 2007. 28(2): p. 183-95.

2. Feng, J., et al., Comprehensive mutation scanning of the dystrophin gene in patients with nonsyndromic X-linked dilated cardiomyopathy. J Am Coll Cardiol, 2002. 40(6): p. 1120-4.

3. Vieitez, I., et al., Mutational spectrum of Duchenne muscular dystrophy in Spain: Study of 284 cases. Neurologia, 2017. 32(6): p. 377-385.

4. Fattahi, Z., et al., Improved diagnostic yield of neuromuscular disorders applying clinical exome sequencing in patients arising from a consanguineous population. Clin Genet, 2017. 91(3): p. 386-402.

5. Xiong, H.Y., et al., RNA splicing. The human splicing code reveals new insights into the genetic determinants of disease. Science, 2015. 347(6218): p. 1254806.

6. Taylor, P.J., et al., Measurement of the clinical utility of a combined mutation detection protocol in carriers of Duchenne and Becker muscular dystrophy. J Med Genet, 2007. 44(6): p. 368-72.

7. Almomani, R., et al., Rapid and cost effective detection of small mutations in the DMD gene by high resolution melting curve analysis. Neuromuscul Disord, 2009. 19(6): p. 383-90.

8. Okubo, M., et al., Comprehensive analysis for genetic diagnosis of Dystrophinopathies in Japan. Orphanet J Rare Dis, 2017. 12(1): p. 149.

9. Li, X., et al., A comprehensive database of Duchenne and Becker muscular dystrophy patients (0-18 years old) in East China. Orphanet J Rare Dis, 2015. 10: p. 5.

10. Juan-Mateu, J., et al., Interplay between DMD point mutations and splicing signals in Dystrophinopathy phenotypes. PLoS One, 2013. 8(3): p. e59916.

11. Babić Božović, I., et al., Diagnostic yield of exome sequencing in myopathies: Experience of a Slovenian tertiary centre. PLoS One, 2021. 16(6): p. e0252953.

12. Tomar, S., et al., Mutational spectrum of dystrophinopathies in Singapore: Insights for genetic diagnosis and precision therapy. Am J Med Genet C Semin Med Genet, 2019. 181(2): p. 230-244.

13. Roberts, R.G., R.J. Gardner, and M. Bobrow, Searching for the 1 in 2,400,000: a review of dystrophin gene point mutations. Hum Mutat, 1994. 4(1): p. 1-11.

14. Kohli, S., et al., Mutation Spectrum of Dystrophinopathies in India: Implications for Therapy. Indian J Pediatr, 2020. 87(7): p. 495-504.

15. Mendell, J.R., et al., Diagnosis of Duchenne dystrophy by enhanced detection of small mutations. Neurology, 2001. 57(4): p. 645-50.

16. Fajkusová, L., et al., Novel dystrophin mutations revealed by analysis of dystrophin mRNA: alternative splicing suppresses the phenotypic effect of a nonsense mutation. Neuromuscul Disord, 2001. 11(2): p. 133-8.

17. Selvatici, R., et al., Ethnicity-related DMD Genotype Landscapes in European and Non-European Countries. Neurol Genet, 2021. 7(1): p. e536.

18. Mah, J.K., et al., A population-based study of dystrophin mutations in Canada. Can J Neurol Sci, 2011. 38(3): p. 465-74.

19. Dolinsky, L.C., R.S. de Moura-Neto, and D.N. Falcão-Conceição, DGGE analysis as a tool to identify point mutations, de novo mutations and carriers of the dystrophin gene. Neuromuscul Disord, 2002. 12(9): p. 845-8.

20. Nigro, V., et al., Detection of a nonsense mutation in the dystrophin gene by multiple SSCP. Hum Mol Genet, 1992. 1(7): p. 517-20.

21. Bennett, R.R., et al., Automated DNA mutation detection using universal conditions direct sequencing: application to ten muscular dystrophy genes. BMC Genet, 2009. 10: p. 66.

22. Zimowski, J.G., et al., Small mutations in Duchenne/Becker muscular dystrophy in 164 unrelated Polish patients. J Appl Genet, 2021. 62(2): p. 289-295.

23. Ashton, E.J., et al., Simultaneous mutation scanning for gross deletions, duplications and point mutations in the DMD gene. Eur J Hum Genet, 2008. 16(1): p. 53-61.

24. Nigro, V., et al., Novel small mutations along the DMD/BMD gene associated with different phenotypes. Hum Mol Genet, 1994. 3(10): p. 1907-8.

25. Wang, L., et al., Genotypes and Phenotypes of DMD Small Mutations in Chinese Patients With Dystrophinopathies. Front Genet, 2019. 10: p. 114.

26. Bai, Y., et al., [Mutation screening of 433 families with Duchenne/Becker muscular dystrophy]. Zhonghua Yi Xue Za Zhi, 2016. 96(16): p. 1261-9.

27. Torella, A., et al., One hundred twenty-one dystrophin point mutations detected from stored DNA samples by combinatorial denaturing high-performance liquid chromatography. J Mol Diagn, 2010. 12(1): p. 65-73.

28. Flanigan, K.M., et al., Rapid direct sequence analysis of the dystrophin gene. Am J Hum Genet, 2003. 72(4): p. 931-9.

29. Whittock, N.V., et al., Dystrophin point mutation screening using a multiplexed protein truncation test. Genet Test, 1997. 1(2): p. 115-23.

30. Tuffery-Giraud, S., et al., The role of muscle biopsy in analysis of the dystrophin gene in Duchenne muscular dystrophy: experience of a national referral centre. Neuromuscul Disord, 2004. 14(10): p. 650-8.

31. Yamputchong, P., et al., Genotype and age at diagnosis in Thai boys with Duchenne muscular dystrophy (DMD). Neuromuscul Disord, 2020. 30(10): p. 839-844.

32. Takeshima, Y., et al., Mutation spectrum of the dystrophin gene in 442 Duchenne/Becker muscular dystrophy cases from one Japanese referral center. J Hum Genet, 2010. 55(6): p. 379-88.

33. Park, H.J., et al., Discovery of pathogenic variants in a large Korean cohort of inherited muscular disorders. Clin Genet, 2017. 91(3): p. 403-410.

34. Ma, P., et al., Comprehensive genetic characteristics of dystrophinopathies in China. Orphanet J Rare Dis, 2018. 13(1): p. 109.

35. Flanigan, K.M., et al., Mutational spectrum of DMD mutations in dystrophinopathy patients: application of modern diagnostic techniques to a large cohort. Hum Mutat, 2009. 30(12): p. 1657-66.

36. Wang, D., et al., Molecular Genetics Analysis of 70 Chinese Families With Muscular Dystrophy Using Multiplex Ligation-Dependent Probe Amplification and Next-Generation Sequencing. Front Pharmacol, 2019. 10: p. 814.

37. Xue, J., et al., [Mutation screening of the dystrophin gene in 14 Chinese Duchenne/Becker muscular dystrophy patients without gross deletions]. Zhonghua Yi Xue Yi Chuan Xue Za Zhi, 2008. 25(6): p. 633-6.

38. Luce, L.N., et al., Small mutation screening in the DMD gene by whole exome sequencing of an argentine Duchenne/Becker muscular dystrophies cohort. Neuromuscul Disord, 2018. 28(12): p. 986-995.

39. Prior, T.W., et al., Identification of two point mutations and a one base deletion in exon 19 of the dystrophin gene by heteroduplex formation. Hum Mol Genet, 1993. 2(3): p. 311-3.

40. Carlson, C.R., S.A. Moore, and K.D. Mathews, Dystrophinopathy muscle biopsies in the genetic testing ERA: One center's data. Muscle Nerve, 2018.

41. Cho, A., et al., Consecutive analysis of mutation spectrum in the dystrophin gene of 507 Korean boys with Duchenne/Becker muscular dystrophy in a single center. Muscle Nerve, 2017. 55(5): p. 727-734.

42. Straathof, C.S., et al., Diagnosis of becker muscular dystrophy: Results of Re-analysis of DNA samples. Muscle Nerve, 2016. 53(1): p. 44-8.

43. Neri, M., et al., The Genetic Landscape of Dystrophin Mutations in Italy: A Nationwide Study. Front Genet, 2020. 11: p. 131.

44. Hofstra, R.M., et al., DGGE-based whole-gene mutation scanning of the dystrophin gene in Duchenne and Becker muscular dystrophy patients. Hum Mutat, 2004. 23(1): p. 57-66.

45. Spitali, P., et al., Exon skipping-mediated dystrophin reading frame restoration for small mutations. Hum Mutat, 2009. 30(11): p. 1527-34.

46. Sironi, M., et al., A novel splice site mutation (3157+1G>T) in the dystrophin gene causing total exon skipping and DMD phenotype. Hum Mutat, 2001. 17(3): p. 239.

47. Bennett, R.R., et al., Detection of mutations in the dystrophin gene via automated DHPLC screening and direct sequencing. BMC Genet, 2001. 2: p. 17.

48. Hua, C., L. Liu, and X. Kong, Prenatal diagnosis of 1408 foetuses at risk of DMD/BMD by MLPA and Sanger sequencing combined with STR linkage analysis. BMC Med Genomics, 2023. 16(1): p. 310.

49. Guo, R., et al., DMD mutation spectrum analysis in 613 Chinese patients with dystrophinopathy. J Hum Genet, 2015. 60(8): p. 435-42.

50. Tong, Y.R., et al., A Comprehensive Analysis of 2013 Dystrophinopathies in China: A Report From National Rare Disease Center. Front Neurol, 2020. 11: p. 572006.

51. Aartsma-Rus, A., et al., Entries in the Leiden Duchenne muscular dystrophy mutation database: an overview of mutation types and paradoxical cases that confirm the reading-frame rule. Muscle Nerve, 2006. 34(2): p. 135-44.

52. Wei, X., et al., Targeted next-generation sequencing as a comprehensive test for patients with and female carriers of DMD/BMD: a multi-population diagnostic study. Eur J Hum Genet, 2014. 22(1): p. 110-8.

53. Lo, I.F., et al., A different spectrum of DMD gene mutations in local Chinese patients with Duchenne/Becker muscular dystrophy. Chin Med J (Engl), 2006. 119(13): p. 1079-87.

54. Adachi, K., et al., [Dystrophin gene analysis on 76 families with dystrophinopathy]. No To Hattatsu, 2002. 34(5): p. 391-7.

55. Tuffery-Giraud, S., et al., Point mutations in the dystrophin gene: evidence for frequent use of cryptic splice sites as a result of splicing defects. Hum Mutat, 1999. 14(5): p. 359-68.

56. de Almeida, P.A.D., et al., Genetic profile of Brazilian patients with dystrophinopathies. Clin Genet, 2017. 92(2): p. 199-203.

57. Kekou, K., et al., Retrospective analysis of persistent HyperCKemia with or without muscle weakness in a case series from Greece highlights vast DMD variant heterogeneity. Expert Rev Mol Diagn, 2023. 23(11): p. 999-1010.

58. López-Hernández, L.B., et al., Comparison of mutation profiles in the Duchenne muscular dystrophy gene among populations: implications for potential molecular therapies. Int J Mol Sci, 2015. 16(3): p. 5334-46.

59. Tay, S.K., et al., Diagnostic strategy for the detection of dystrophin gene mutations in asian patients and carriers using immortalized cell lines. J Child Neurol, 2006. 21(2): p. 150-5.

60. Chen, C., et al., Screening of Duchenne muscular dystrophy (DMD) mutations and investigating its mutational mechanism in Chinese patients. PLoS One, 2014. 9(9): p. e108038.

61. Prior, T.W., et al., Spectrum of small mutations in the dystrophin coding region. Am J Hum Genet, 1995. 57(1): p. 22-33.

62. Mercier, S., et al., Genetic and clinical specificity of 26 symptomatic carriers for dystrophinopathies at pediatric age. Eur J Hum Genet, 2013. 21(8): p. 855-63.

63. Long, F., et al., [Clinical application of multiplex ligation-dependent probe amplification for the detection exonic copy number alterations in the Dystrophin gene]. Zhonghua Yi Xue Yi Chuan Xue Za Zhi, 2011. 28(6): p. 699-704.

64. Hwa, H.L., et al., Small mutations of the DMD gene in Taiwanese families. J Formos Med Assoc, 2008. 107(6): p. 463-9.

65. Brogna, C., et al., The nonsense mutation stop+4 model correlates with motor changes in Duchenne muscular dystrophy. Neuromuscul Disord, 2021. 31(6): p. 479-488.

66. Xiao, T., et al., Genetic identification of pathogenic variations of the DMD gene: a retrospective study from 10,481 neonatal patients based on next-generation sequencing data. Ann Transl Med, 2021. 9(9): p. 766.

67. Nallamilli, B.R.R., et al., A single NGS-based assay covering the entire genomic sequence of the DMD gene facilitates diagnostic and newborn screening confirmatory testing. Hum Mutat, 2021. 42(5): p. 626-638.

68. Roest, P.A., et al., Application of in vitro Myo-differentiation of non-muscle cells to enhance gene expression and facilitate analysis of muscle proteins. Neuromuscul Disord, 1996. 6(3): p. 195-202.

69. Liu, M.J., et al., [Application of next-generation sequencing technology for genetic diagnosis of Duchenne muscular dystrophy]. Zhonghua Yi Xue Yi Chuan Xue Za Zhi, 2012. 29(3): p. 249-54.

70. Hamed, S.A. and E.P. Hoffman, Automated sequence screening of the entire dystrophin cDNA in Duchenne dystrophy: point mutation detection. Am J Med Genet B Neuropsychiatr Genet, 2006. 141b(1): p. 44-50.

71. Zhang, J., et al., Genetic analysis of 62 Chinese families with Duchenne muscular dystrophy and strategies of prenatal diagnosis in a single center. BMC Med Genet, 2019. 20(1): p. 180.

72. Suárez-Calvet, X., et al., Decoding the transcriptome of Duchenne muscular dystrophy to the single nuclei level reveals clinical-genetic correlations. Cell Death Dis, 2023. 14(9): p. 596.

73. Wang, H., et al., Prenatal diagnosis of Duchenne muscular dystrophy in 131 Chinese families with dystrophinopathy. Prenat Diagn, 2017. 37(4): p. 356-364.

74. Kong, X., et al., Genetic analysis of 1051 Chinese families with Duchenne/Becker Muscular Dystrophy. BMC Med Genet, 2019. 20(1): p. 139.

75. Hu, X., et al., Proband-only medical exome sequencing as a cost-effective first-tier genetic diagnostic test for patients without prior molecular tests and clinical diagnosis in a developing country: the China experience. Genet Med, 2018. 20(9): p. 1045-1053.

76. Marinakis, N.M., et al., Phenotype-driven variant filtration strategy in exome sequencing toward a high diagnostic yield and identification of 85 novel variants in 400 patients with rare Mendelian disorders. Am J Med Genet A, 2021. 185(8): p. 2561-2571.

77. Xie, Z., et al., Splicing Characteristics of Dystrophin Pseudoexons and Identification of a Novel Pathogenic Intronic Variant in the DMD Gene. Genes (Basel), 2020. 11(10).

78. Lesca, G., et al., [Symptomatic carriers of dystrophinopathy with chromosome X inactivation bias]. Rev Neurol (Paris), 2003. 159(8-9): p. 775-80.

79. Yang, Y.M., et al., Comprehensive genetic diagnosis of patients with Duchenne/Becker muscular dystrophy (DMD/BMD) and pathogenicity analysis of splice site variants in the DMD gene. J Zhejiang Univ Sci B, 2019. 20(9): p. 753-765.

80. Bartolo, C., et al., A novel splice site mutation in a Becker muscular dystrophy patient. J Med Genet, 1996. 33(4): p. 324-7.

81. Zhong, J., et al., Genetic analysis of the dystrophin gene in children with Duchenne and Becker muscular dystrophies. Muscle Nerve, 2017. 56(1): p. 117-121.

82. Oliveira, R., et al., Inherited myopathies in patients from Sub-Saharan Africa: Results from a retrospective cohort. J Clin Neurosci, 2022. 106: p. 43-48.

83. Tian, P.C., et al., [Application of next-generation sequencing in the molecular diagnosis of Duchenne muscular dystrophy]. Zhongguo Dang Dai Er Ke Za Zhi, 2019. 21(3): p. 244-248.

84. Roberts, R.G., M. Bobrow, and D.R. Bentley, Point mutations in the dystrophin gene. Proc Natl Acad Sci U S A, 1992. 89(6): p. 2331-5.

85. Dent, K.M., et al., Improved molecular diagnosis of dystrophinopathies in an unselected clinical cohort. Am J Med Genet A, 2005. 134(3): p. 295-8.

86. Daoud, F., et al., Analysis of Dp71 contribution in the severity of mental retardation through comparison of Duchenne and Becker patients differing by mutation consequences on Dp71 expression. Hum Mol Genet, 2009. 18(20): p. 3779-94.

87. Zimowski, J.G., et al., [Detection of rare mutations in the dystrophin gene]. Med Wieku Rozwoj, 2009. 13(2): p. 140-5.

88. Kumar, S.H., et al., Comprehensive genetic analysis of 961 unrelated Duchenne Muscular Dystrophy patients: Focus on diagnosis, prevention and therapeutic possibilities. PLoS One, 2020. 15(6): p. e0232654.

89. Lenk, U., et al., Point mutations at the carboxy terminus of the human dystrophin gene: implications for an association with mental retardation in DMD patients. Hum Mol Genet, 1993. 2(11): p. 1877-81.

90. Tuffery, S., et al., Mutation analysis of the dystrophin gene in Southern French DMD or BMD families: from Southern blot to protein truncation test. Hum Genet, 1998. 102(3): p. 334-42.

91. Comi, G.P., et al., A G+1-->A transversion at the 5' splice site of intron 69 of the dystrophin gene causing the absence of peripheral nerve Dp116 and severe clinical involvement in a DMD patient. Hum Mol Genet, 1995. 4(11): p. 2171-4.

92. Barbieri, A.M., et al., Seven novel additional small mutations and a new alternative splicing in the human dystrophin gene detected by heteroduplex analysis and restricted RT-PCR heteroduplex analysis of illegitimate transcripts. Eur J Hum Genet, 1996. 4(3): p. 183-7.

93. Lasa, A., P. Gallano, and M. Baiget, Three novel point mutations in the dystrophin gene in DMD patients. Hum Mutat, 1997. 9(5): p. 473-4.
